# Targeting the KLF5-EphA2 axis can restrain cancer stemness and overcome chemoresistance in basal-like breast cancer

**DOI:** 10.7150/ijbs.82567

**Published:** 2023-03-21

**Authors:** Ping Zhao, Jian Sun, Xinwei Huang, Xiangwu Zhang, Xin Liu, Rong Liu, Guangshi Du, Wenqiang Gan, Chuanyu Yang, Yiyin Tang, Ceshi Chen, Dewei Jiang

**Affiliations:** 1 The Third Affiliated Hospital, Kunming Medical University, Kunming, 650118 China; 2 Key Laboratory of The Second Affiliated Hospital of Kuming Medical College, Kunming, 650101, China; 3 Translational Cancer Research Center, Peking University First Hospital, Beijing, 100034 China; 4 Translational Medicine Research Center, Guizhou Medical University, Guiyang, 550025 China; 5 Key Laboratory of Animal Models and Human Disease Mechanisms of the Chinese Academy of Sciences and Yunnan Province, Kunming Institute of Zoology, Chinese Academy of Sciences, Kunming, 650201 China; 6 Kunming College of Life Sciences, University of the Chinese Academy of Sciences, Kunming, 650204 China; 7 Academy of Biomedical Engineering, Kunming Medical University, Kunming, 650500 China

**Keywords:** EphA2, TNF-α, KLF5, BLBC stem cells, Chemoresistance, ALW-II-41-27

## Abstract

Ephrin type-A receptor 2 (EphA2) is a member of the tyrosine receptor kinases, a family of membrane proteins recognized as potential anticancer targets. EphA2 highly expressed in a variety of human cancers, playing roles in proliferation, migration, and invasion. However, whether and how EphA2 regulates basal-like breast cancer (BLBC) cell stemness and chemoresistance has not been revealed. Here, KLF5 was proven to be a direct transcription factor for *EphA2* in BLBC cells, and its expression was positively correlated in clinical samples from breast cancer patients. The inflammatory factor TNF-α could promote BLBC cell stemness partially by activating the KLF5-EphA2 axis. Moreover, phosphorylation of EphA2 at S897 (EphA2 pS897) induced by TNF-α and PTX/DDP contributes to chemoresistance of BLBC. Furthermore, the EphA2 inhibitor ALW-II-41-27 could effectively reduce EphA2 pS897 and tumor cell stemness *in vitro* and significantly enhance the sensitivity of xenografts to the chemotherapeutic drugs PTX and DDP *in vivo*. Clinically, tumor samples from breast patients with less response to neoadjuvant chemotherapy showed a high level of EphA2 pS897 expression. In conclusion, KLF5-EphA2 promotes stemness and drug resistance in BLBC and could be a potential target for the treatment of BLBC.

## Introduction

As the most common malignant tumor, breast cancer poses a great threat to women's health worldwide^1^. Breast cancer is a highly heterogeneous group of tumors, of which basal-like breast cancer (BLBC) is a type of most malignant breast cancer, accounting for 10-20% of all breast cancers^2^. It is well known that BLBC is characterized by high heterogeneity, poor prognosis, and susceptibility to recurrence and metastasis^3^ and is mainly treated through chemotherapy. Approximately 20% of patients with BLBC achieve pathological complete remission (pCR) after treatment with chemotherapeutic drugs such as taksol, anthracycline, and platinum^4^, while more BLBC patients experienced treatment failure. Therefore, it is urgent to explore the mechanism of chemotherapy resistance and identify effective therapeutic targets^5^.

Currently, it is generally recognized that tumor stem cells are responsible for the occurrence and progression of tumors. A group of cells with high expression of CD44^high^/CD24^low^ or ALDH^+^ in breast cancer are considered to be breast cancer stem cells (BCSC)^6^. It was noted that in clinical samples of breast cancer, ALDH^+^ accounted for 39.4% in samples of BLBC, and similar results were obtained in studies on breast cancer cell lines^7^, while lower expression was observed in intraductal carcinoma and luminal type with better prognosis^8^. It has been demonstrated that BCSCs are closely related to BLBC invasion^9^, metastasis^10^, and drug resistance^11^-^13^, and chemotherapy resistance resulting from BCSCs is considered to be attributed to high recurrence and metastasis of BLBC^14^, ^15^. Therefore, BLBC treatment failure due to chemotherapy resistance is closely related to the characteristics of tumor stem cells.

Moreover, tumorigenesis and cancer progression are also linked to the inflammatory environment^16^. Tumor necrosis factor (TNF-α), a widely known inflammatory factor, plays an important role in the tumor microenvironment. TNF-α can induce breast cancer stem cells by upregulating Slug through NF-κB/HIF1α^17^. As previously reported, TNF-α induces TAZ expression through the nonclassical NF-κB pathway to promote breast cancer cell stemness and induces the expression of the transcription factor KLF5 in BLBC cells^18^, ^19^. KLF5 is highly expressed in BLBC and is associated with poor prognosis^20^, stemness^21^ and metastasis^22^. KLF5, together with Oct4, c-Myc, and Sox2, can induce differentiated mouse fibroblasts into pluripotent stem cells^23^. In addition, knockdown or inhibition of KLF5 expression with metformin and mifepristone derivatives could reduce the proportion of tumor stem cells in BLBC^24^, ^25^. Collectively, our studies have indicated that KLF5 is a key transcription factor that promotes the stemness and progression of BLBC^26^.

Ephrin type-A receptor 2 (EphA2), a member of the receptor tyrosine kinase (RTK) family, is a type I transmembrane glycoprotein. Increasing evidence has shown that EphA2 is highly expressed in a variety of human cancers, such as breast cancer, prostate cancer, lung cancer, colon cancer, and skin cancer^27^-^31^. EphA2 activation affects the phosphorylation levels of JNK and c-JUN, which promotes stemness in non-small cell lung cancer, thus promoting proliferation and metastasis^32^. Phosphorylation of EphA2 at S897 mediates the activation of the AKT, STAT3, SOX-2, and c-MYC signaling pathways, which is crucial to nasopharyngeal carcinoma stem cell formation^33^. EphA2 has been reported to play an important role in the proliferation, migration, and invasion^34^-^37^ of BLBC. However, it remains unclear whether EphA2 is associated with BLBC cell stemness and chemotherapy resistance.

In this study, we first revealed that KLF5, an important BLBC stem cell regulator, promoted* EphA2* expression and that TNF-α promoted BLBC cell stemness and drug resistance by activating the KLF5-EphA2 axis. Moreover, EphA2 pS897 induced by TNF-α and PTX/DDP mediated chemo-resistance of BLBC. ALW-II-41-27 could effectively reduce EphA2 pS897 and increase the chemotherapy sensitivity of BLBC cells. Thus, KLF5-EphA2 promotes stemness and drug resistance in BLBC, providing new insights into therapeutics against BLBC.

## Results

### Knockdown of EphA2 attenuates the stemness of BLBC cells

**Figure 1:**
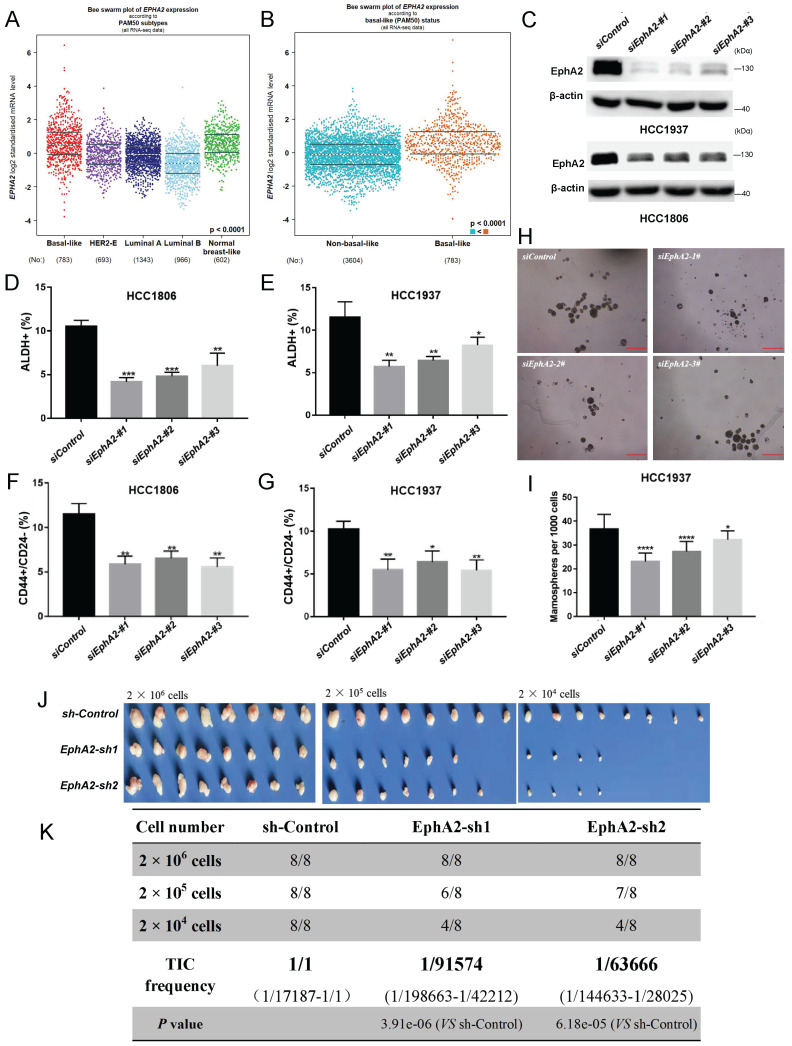
**EphA2 is highly expressed in BLBC and promotes breast cancer cell stemness. (A)** According to database analysis (bc-GenExMiner v4.9), EphA2 mRNA levels were found to be highly expressed in basal-like breast cancers (BLBC); **(B)** According to TCGA database analysis, EphA2 mRNA levels were found to be highly expressed in BLBC patients; **(C-I)** After knockdown of EphA2 in HCC1937 and HCC1806 (C), flow detection of ALDH^+^ (D-E) and CD44^+^/CD24^-^(F-G) cell ratios and mamosphere formation ratio was performed (H-I), Scale bar=250 μm; **(J-K)** Dilution tumorigenesis assay in nude mice (J), tumorigenesis rate statistics table (K).

Through clinical analysis, EphA2 was highly expressed in BLBC patients or triple-negative breast cancer (TNBC) patients based on DNA microarray data (Figure 1A-B, and Figure S1A) or TCGA data (Figure S1B), and its high expression indicated a poor prognosis in breast cancer patients (Figure S1C). To explore whether EphA2 was associated with breast cancer cell stemness, EphA2 was knocked down by three independent siRNAs in two BLBC cell lines, HCC1937 and HCC1806 (Figure 1C). Flow cytometry analysis showed that the proportion of either ALDH^+^ cells or CD44^high^/CD24^low^ cells, two well-recognized markers for BCSCs, was significantly reduced in *EphA2*-deficient cells (Figure 1D-G). Consistently, knockdown of *EphA2* resulted in a significant decrease in the number of tumorspheres in HCC1937 cells (Figure 1H-I). In addition, *EphA2* expression was correlated with that of three *ALDH1* variants, especially *ALDH1A3,* and several CSC-related genes, including *NOTCH1*, *EGFR*, and *FOSL1* (Figure S1D-F). Further* in vivo* experiments on the xenograft transplantation mouse model with limiting dilution HCC1806 cells revealed remarkably lower frequencies of tumor formation upon *EphA2* knockdown (Figure 1J-K and Figure S1G). These data suggested that EphA2 could function in stemness maintenance of BLBC cells.

### KLF5 is a transcription factor of EphA2 and coexpressed in BLBC cells and clinical specimens

**Figure 2:**
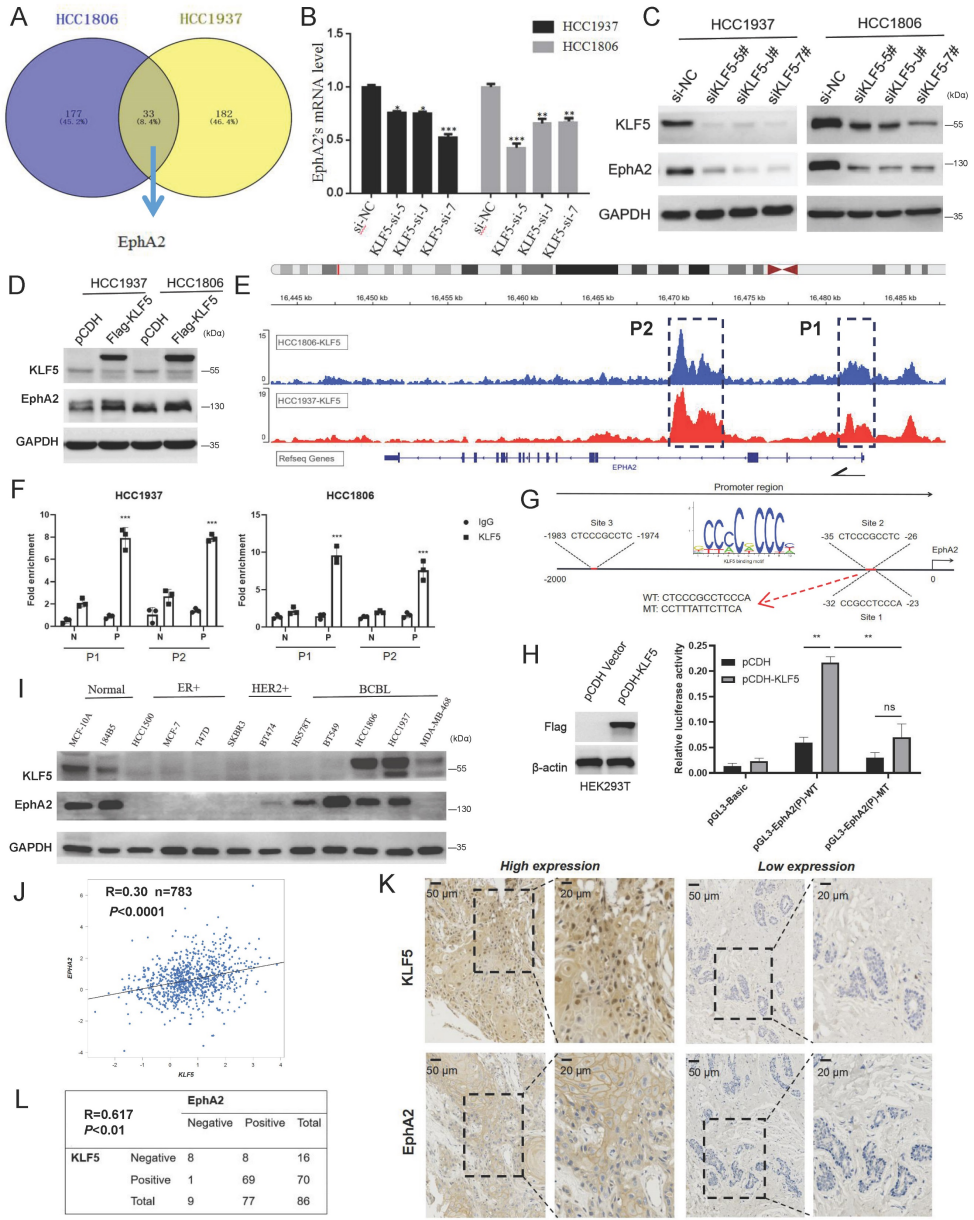
** KLF5 positively regulates EphA2 expression in BLBC. (A)** Knockdown of KLF5 in HCC1937 and HCC1806 cells for RNA-seq and 33 genes were found to be positively regulated by KLF5, including EphA2. **(B-C)** Knockdown of KLF5 was confirmed to suppress EphA2 expression by RT‒qPCR and WB assay; **(D)** Overexpression of KLF5 promotes EphA2 expression by WB assay; **(E)** ChIP-seq results of KLF5 in HCC1937 and HCC1806 cells showed two potential binding sites of KLF5 in the *EphA2* gene locus, which were named P1 and P2, respectively; **(F)** Validation of KLF5 binding sites P1 and P2 in the* EphA2* gene by ChIP‒qPCR in HCC1937 and HCC1806, respectively. P (Positive) or N (Negative) is the primer targeting P1/P2 or distant non-peak sequence, respectively. **G**. JASPAR database analysis of the KLF5 binding motif and potential binding sites of KLF5 in the promoter region of EphA2 (Site 3 is out of P1/P2 and Sites 1/2 share common motif); **(H)** Luciferase activity assay for detecting the transcriptional activity of WT and mutated promoters of *EphA2* in HEK293T cells in the absence or presence of KLF5 overexpression. **(I)** In multiple breast cancer cell lines and normal breast epithelial cell lines, a WB assay found a certain trend of coexpression of KLF5 and EphA2; **(J)** Database (bc-GenExMiner v4.9) analysis revealed a correlation between the mRNA expression of *EphA2* and *KLF5* in BLBC patients (n=783) (R=0.3); **(K-L)** Detection by tissue microarray in 86 cases revealed that EphA2 and KLF5 were both highly expressed in BLBC and that their expression was positively correlated.

We have long focused on the function of the transcription factor KLF5 in regulating BLBC stemness^24^, ^25^ which is of high expression in BLBC and indicated poor prognosis in high grade breast cancer patients (Figure S2A-C), and found that *EphA2* may be positively regulated by KLF5 based on the transcriptome analysis of shKLF5/shCon HCC1806 and HCC1937 cells (Figure 2A). *KLF5* knockdown decreased the mRNA level of *EphA2* (Figure 2B), and its protein expression could be down- or upregulated by *KLF5* knockdown or overexpression, respectively (Figure 2C-D), while *EphA2* knockdown did not change KLF5 expression (Figure S2D). Based on our KLF5 chromatin immunoprecipitation (ChIP)-sequencing data^38^, enrichment of KLF5 binding peaks was found in two regions of the *EphA2* locus, one around the first exon (P1) and another in the third intron (P2) (Figure 2E). ChIP‒qPCR assays confirmed that the KLF5 antibody, but not the control IgG, specifically pulled down the predicted P1 and P2 fragments (Figure 2F). However, potential KLF5 binding sites were only predicted in P1 (Figure 2G), indicating indirect binding of KLF5 to P2. A dual luciferase reporter assay in HEK293T cells showed that, compared with the control vector, KLF5 overexpression significantly activated P1-mediated luciferase expression, which was abolished when the binding site was mutated (Figure 2H). These data suggested that KLF5 binds to the *EphA2* promoter to activate its expression in BLBC cells.

Moreover, EphA2 and KLF5 proteins were coexpressed in immortalized breast epithelial cell lines (MCF10A and 184B5) and BLBC cell lines (HCC1937 and HCC1806) (Figure 2I). In contrast, both were expressed at low levels in ER- and HER2-positive breast cancer cell lines (HCC1500, MCF-7, T47D, SKBR3, and BT474) (Figure 2I). Analysis by the breast cancer database (bc-GenExMiner v4.5) showed a positive correlation between *EphA2* and *KLF5* in basal-like patients (R=0.30, n=783) (Figure 2J) and all breast cancer patients (R=0.36, n=4421) (Figure S2E). Consistently, the expression correlation between *EphA2* and two established KLF5 targets, *FGF-BP1* and *KRT16,* was observed (Figure S2E). A similar correlation was also observed via analyses of TCGA breast cancer data (Figure S2F) or GTEx normal mammary data (Figure S2G). Consistently, immunohistochemical (IHC) detection further showed joint positive staining of KLF5 and EphA2 in 80.2% of BLBC clinical specimens (Figure 2K-L). These results suggest that EphA2 and KLF5 are coexpressed in BLBC cells and clinical specimens.

### TNF-α induces stemness of BLBC cells partially by activating the KLF5-EphA2 axis

**Figure 3:**
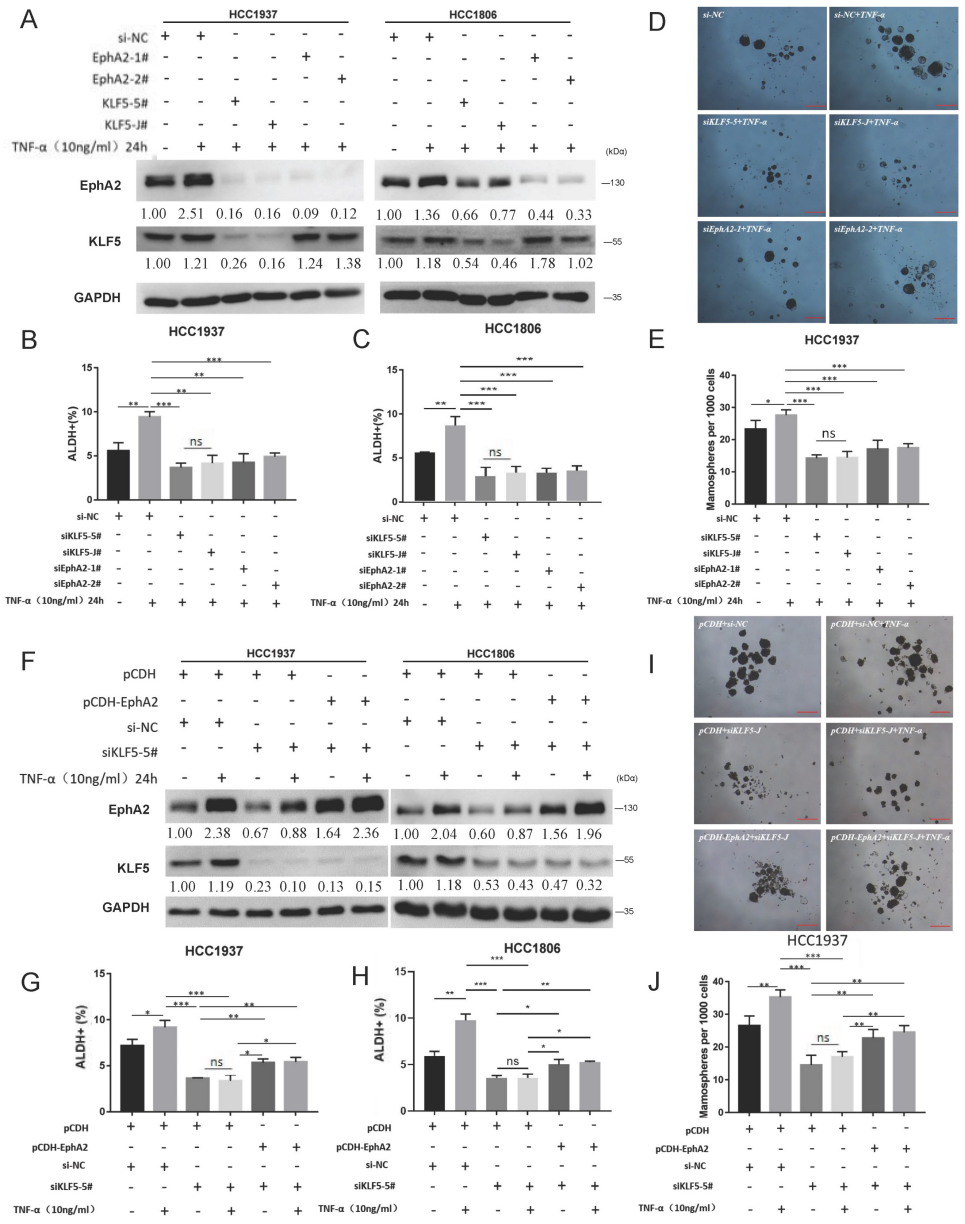
** TNF-α induces stemness of BLBC cells partially by activating KLF5-EphA2. (A-E)** Knockdown of EphA2 and KLF5 in HCC1937 and HCC1806, respectively, with concomitant addition of TNF-α for WB assay of EphA2 and KLF5 expression (A), ALDH^+^ cell ratio by flow assay (B-C), Detection of the sphere-forming rate of HCC1937 by microsphere-formation assay (D-E), Scale bar=250 μm; **(F-J)** Knockdown of KLF5 and reversion to overexpression of EphA2 and addition of TNF-α in HCC1937 and HCC1806 for WB detection of EphA2 and KLF5 expression (F), ALDH^+^ cell ratio by flow assay (G-H), Detection of the sphere-forming rate of HCC1937 by mamosphere formation assay (I-J), Scale bar=250 μm. Changed folds in (A) and (F) of indicated proteins were referenced to Control (siNC or pCDH), normalized to GAPDH, respectively.

We and other researchers have reported that TNF-α could promote the stemness of breast cancer cells and induce KLF5 expression in BLBC cells^18^, ^19^. It was speculated that TNF-α could induce the expression of the KLF5-EphA2 axis to promote cancer stemness. To this end, cell stemness was evaluated post TNF-α treatment on BLBC cells with KLF5 or EphA2 knockdown and control cells. As expected, TNF-α treatment stimulated the expression of both KLF5 and EphA2, while *KLF5* knockdown abolished the TNF-α-induced EphA2 increase (Figure 3A). Consistently, TNF-α treatment increased tumorsphere formation (Figure 3B-C and Figure S3) and the ALDH^+^ proportion (Figure 3D-E), which was highly inhibited by either KLF5 or EphA2 knockdown in HCC1937 and/or HCC1806 cells. Further rescue experiments showed that re-expressing EphA2 in KLF5-knockdown cells (Figure 3F), partially but significantly, restored the decrease in the ALDH^+^ proportion (Figure 3G-H) and tumorsphere formation (Figure 3I-J) induced by KLF5 knockdown in HCC1937 and/or HCC1806 cells. Together, the KLF5-EphA2 axis could play an important role in TNF-α-induced BLBC cell stemness.

### Inhibiting EphA2 phosphorylation by ALW-II-41-27 impedes BLBC cell stemness and chemoresistance

**Figure 4:**
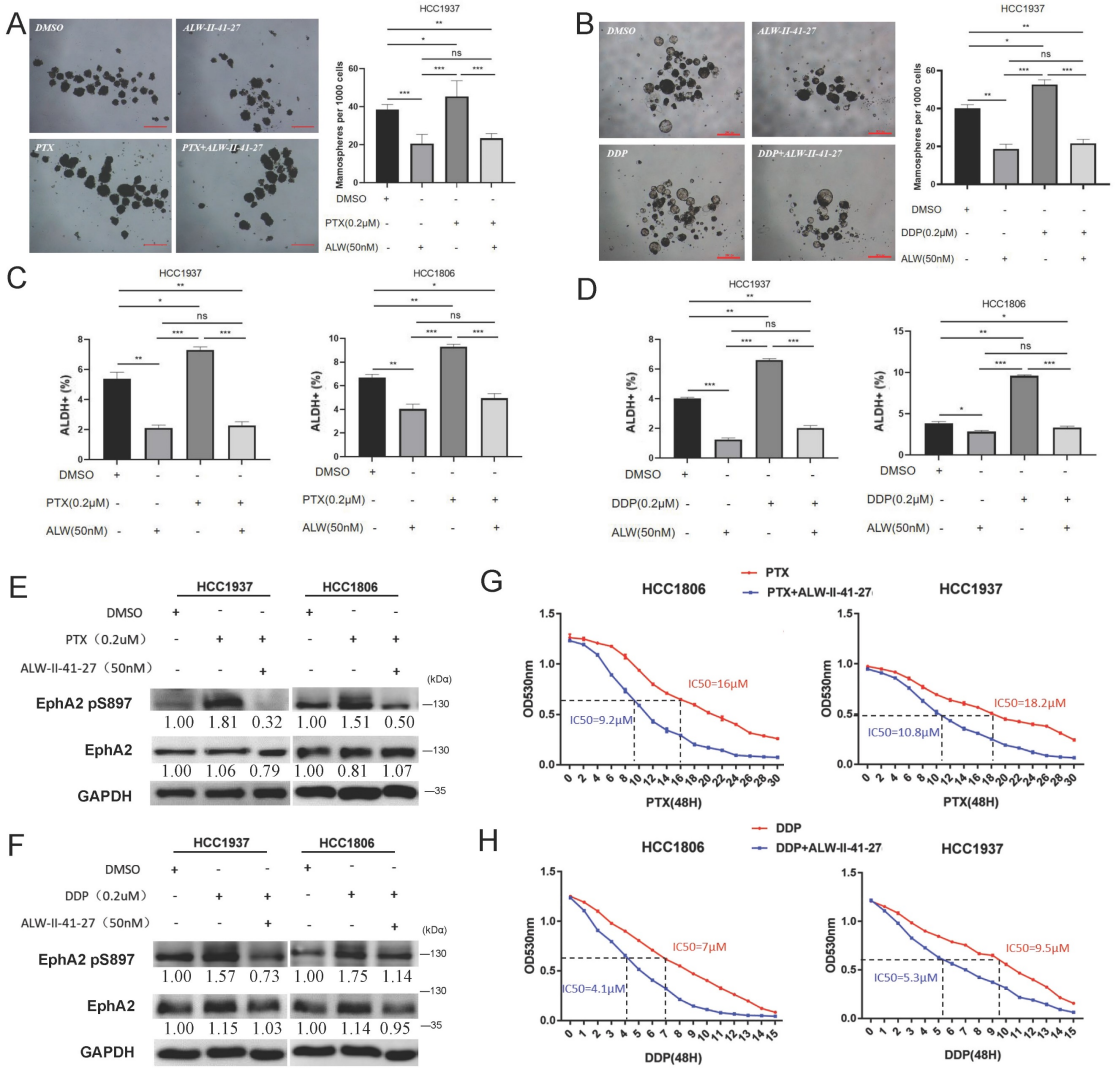
** Inhibiting EphA2 phosphorylation by ALW-II-41-27 impedes BLBC cell stemness and chemoresistance. (A-B)** Detection of sphere formation by mamosphere formation assay after treating HCC1937 cells with chemotherapeutic drugs PTX (2 μM) and DDP (2 μM) alone or in combination with ALW-II-41-27 (50 nM) for 48 h. Scale bar=250 μm. **(C-D)** Detection of the ALDH^+^ cell ratio by flow cytometry after treatment of HCC1937 and HCC1806 cells with the chemotherapeutic agents PTX (2 μM) or DDP (2 μM) alone or in combination with ALW-II-41-27 (50 nM) for 48 h; **(E-F)** Detection of EphA2 and EphA2 pS897 expression by WB after treatment of HCC1937 and HCC1806 cells with the chemotherapeutic agents PTX (2 μM) or DDP (2 μM) alone or in combination with ALW-II-41-27 (50 nM) for 48 h; **(G-H)** IC50 assay in HCC1937 and HCC1806 cells treated with chemotherapeutic agents PTX or DDP alone or in combination with ALW-II-41-27. Changed folds in (E) and (F) of indicated proteins were referenced to DMSO, normalized to GAPDH, respectively.

Cancer stem cells are closely related to chemoresistance^39^-^41^. Here, it was observed that treatment with paclitaxel (PTX) and cisplatinum (DDP) at low doses promoted tumor sphere formation (Figure 4A-B) and the ALDH^+^ proportion (Figure 4C-D). In addition, knockdown of either KLF5 or EphA2 increased the sensitivity of HCC1806 and HCC1937 cells to PTX and DDP (Figure S4A-B). It has been reported that inhibition of EphA2 by ALW-II-41-27 reverses TKI resistance in lung cancer cells. To test whether ALW-II-41-27 also influences BLBC cell stemness and drug sensitivity, we measured the tolerance curve of HCC1806 and HCC1937 cells against ALW-II-41-27 (Figure S4C), and a concentration below IC50 (50 nM) was chosen for subsequent experiments to avoid significant cytotoxicity. As expected, ALW-II-41-27 significantly inhibited the enrichment of tumorsphere and ALDH^+^ cells post PTX and DDP treatment (Figure 4A-D).

Given that ALW-II-41-27 is a highly efficient inhibitor of phosphorylation, but not expression, of EphA2^42^, phosphorylation was supposed to contribute to EphA2-mediated BLBC cell stemness and chemoresistance. Phosphorylation of EphA2, especially at S897, was reported to function in regulating its stability, activation, and function^33^, ^43^. TNF-α was also reported to activate the RSK1/2-EphA2 pS897 axis and promote the migration and invasion of breast cancer cells^44^. Both PTX/DDP and TNF-α treatment significantly increased the EphA2 pS897 level in the two cell lines, which could be largely suppressed by ALW-II-41-27 (Figure 4E-F and Figure S4D). As expected, ALW-II-41-27 significantly sensitized two BLBC cell lines to PTX or DDP treatment, as indicated by a more than 40% decrease in the IC50 value (Figure 4G-H).

### EphA2 pS897 contributes to TNF-α-induced chemoresistance of BLBC cells and is related to the clinical response to neoadjuvant chemotherapy

**Figure 5:**
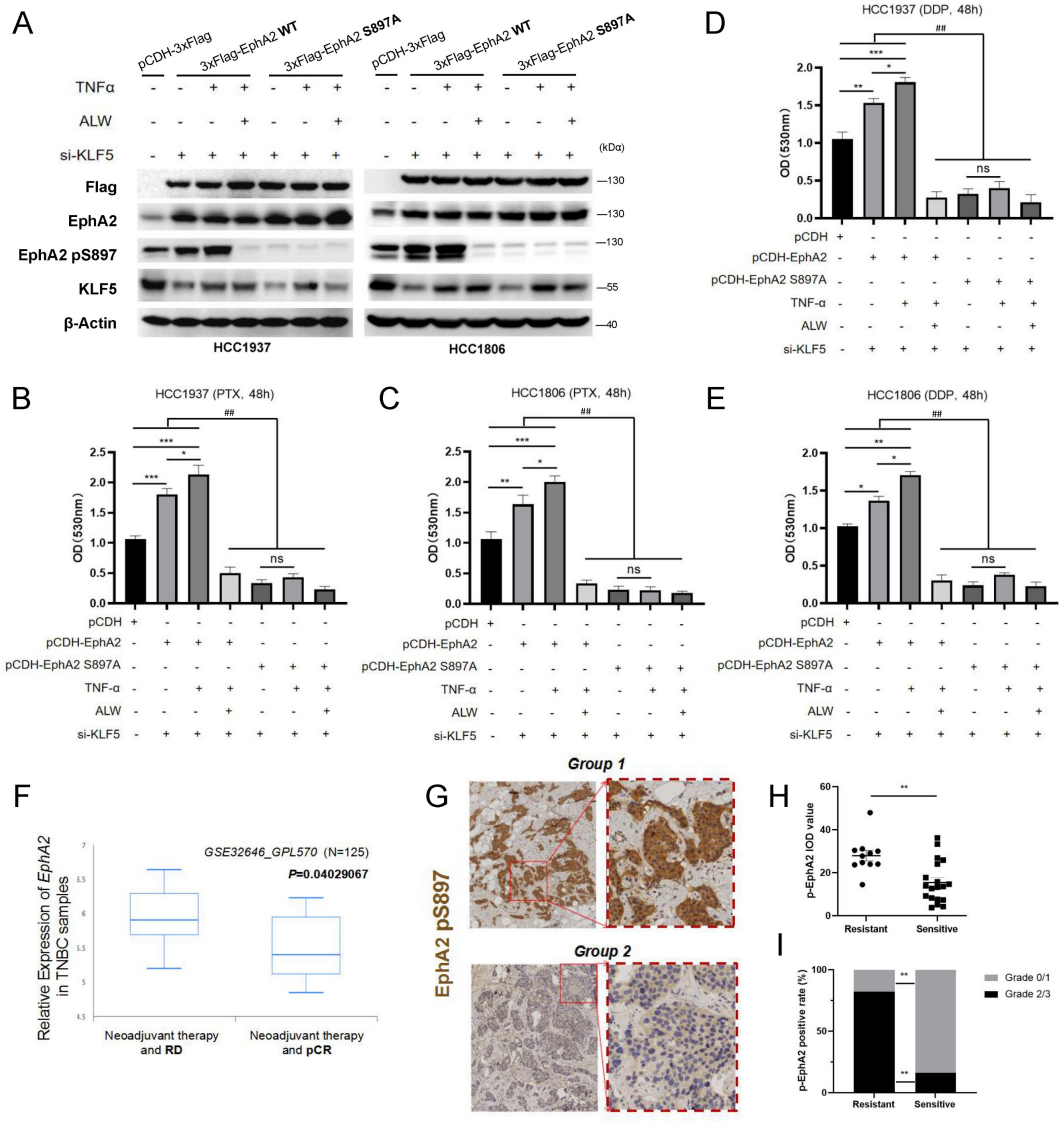
** EphA2 pS897 contributes to BLBC cell chemoresistance and indicates the NCT response. (A)** Detection of EphA2, KLF5 and EphA2 pS897 expression by WB after knocking down KLF5 in HCC1937 and HCC1806 cells while overexpressing EphA2 WT or EphA2 S897A and adding TNF-α(10 ng/mL) and ALW-II-41-27 (ALW; 50 nM); **(B-E)** Detection of the proliferation of HCC1806 and HCC1937 cells under PTX (B-C) or DDP (D-E) treatment. **(F)** *EphA2* expression in samples from BLBC patients (BCIP database) with pCR and residual disease (RD) post neoadjuvant chemotherapy (NCT). **(G)** IHC detection of EphA2 pS897 expression in neoadjuvant chemoresistant (Group 1, N=11) and sensitive (Group 2, N=19) breast cancer tissue samples; **(H-I)** Statistics of IOD score (H) and pathological grading (I) for samples from (G). * or ns indicates the significance between the two and **#** indicates the significance between any one of each groups.

To further characterize the role of EphA2 pS897, we re-expressed wild-type (WT) and mutated EphA2 (S897A) in HCC1937 and HCC1806 cells post-knockdown of *KLF5* (Figure 5A). TNF-α successfully induced pS897 signaling on WT EphA2 but failed on the S897A mutant (Figure 5A). Consistently, the decrease in PTX/DDP tolerance in *KLF5* knockdown cells was restored by re-expressing WT EphA2 but not the S897A mutant (Figure 5B-E). These data revealed that EphA2 pS897 could function as an important downstream effector in TNF-α-KLF5-mediated chemoresistance.

Clinically, analysis of a cohort (GSE32646_GPL570) expectedly showed that *EphA2* expression in samples from BLBC patients with pCR post neoadjuvant chemotherapy (NCT) was lower than that of BLBC patients with residual disease (RD) post NCT (Figure 5F). Based on the function of EphA2 pS897 in BLBC cell chemoresistance, the clinical relevance of EphA2 pS897 to patient outcomes was analyzed. IHC staining of BLBC clinical specimens from breast cancer patients with NCT showed that high expression of EphA2 pS897 was more frequent in chemoresistant samples than in chemosensitive samples (Figure 5G-H). These results suggested that EphA2 pS897 could be used as a potential predictor for the response of breast cancer patients to NCT.

### ALW-II-41-27 enhanced the effect of chemotherapy against BLBC tumors *in vivo*

**Figure 6:**
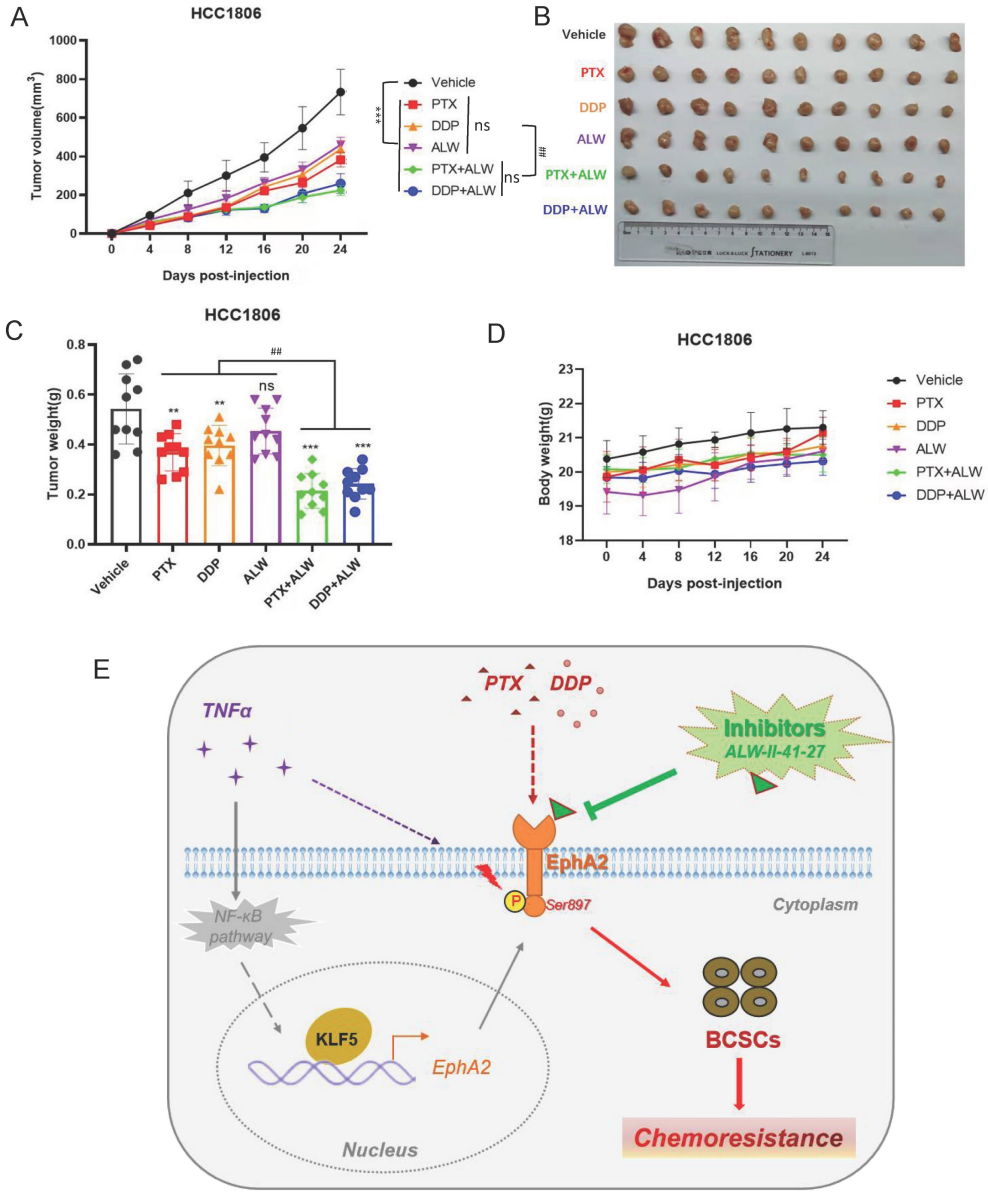
** ALW-II-41-27 enhanced the *in vivo* effect of chemotherapy against BLBC tumors. (A)** Measurement of changes in the size of the tumor in different groups of treatment* in vivo* after treatment with chemotherapeutic agents PTX (1 mg/kg) or DDP (1 mg/kg), alone or in combination with ALW-II-41-27 (1 mg/kg); **(B)** Display of tumor tissues removed at the endpoint of control or drug treatment (day 24); **(C)** Statistics for weight of all tumor tissues; **(D)** Body weight of nude mice in each group; **(E)** Schematic representation of the scientific hypothesis that the inflammatory factor TNF-α promotes BLBC cell stemness by activating KLF5-EphA2 axis expression and thereby promoting pS897 EphA2-mediated chemoresistance; EphA2 inhibitor ALW-II-41-27 inhibits BLBC cell stemness and enhances chemotherapeutic drug sensitivity of BLBC cells, indicating that the membrane protein EphA2 is a potential therapeutic target for BLBC. * or ns indicates the significance between Viechle group and other individual group, and **#** indicates the significance between single drug groups (PTX, DDP, or ALW) with combined-drugs groups (DDP+ALW, or PTX+ALW).

Next, we further tested whether ALW-II-41-27 could enhance the efficacy of PTX or DDP treatment in a tumor-bearing nude mouse model established with HCC1806 cells. ALW-II-41-27 showed a favorable inhibitory effect in the cell experiments at a low concentration (50 nM); thus, a lower dose of ALW-II-41-27 (1 mg/kg) and a lower dose of PTX (0.75 mg/kg) or DDP (0.5 mg/kg) were used on mice to reduce chemotoxicity as much as possible. Administration of PTX or DDP inhibited tumor growth to some extent, while ALW-II-41-27 alone showed little inhibition of tumor growth (Figure 6A-C). Interestingly, the combination of ALW-II-41-27 and PTX or DDP dramatically deceased tumor growth, much more efficiently than that of a single drug (Figure 6A-C). Noticeably, as expected, all mice showed no loss of body weight, suggesting no significant toxicity of the drug combination (Figure 6D). These results suggested that ALW-II-41-27 successfully enhanced the chemotherapeutic effect of PTX and DDP at low doses, which has positive significance for the principle of "reduction and efficiency" of clinical drugs.

## Discussion

KLF5 functions as a cancer-promoting transcription factor in a variety of cancers, including breast, bladder, and intestinal cancers. Previous studies have demonstrated that KLF5 promotes BLBC cell stemness, proliferation, migration, and invasion through transcriptional regulation of *FGF-BP1*, *TNFAIP2*, *Slug*, *Cyclin D*1, and other gene expression^25^, ^45^-^47^. In this study, we identified for the first time that *EphA2* is another direct target of KLF5 in BLBC. Increasing evidence suggests that EphA2 expression is closely related to poor prognosis, increased metastatic potential, and reduced survival in tumor patients^48^, ^49^. Thus, the close correlation of EphA2 expression and function with malignancy makes this protein an important target for cancer therapy. It was confirmed through sphere-forming assay, flow cytometry and dilution tumorigenesis experiments on BLBC cells or a mouse model that EphA2 exhibited similar roles in promoting the stemness of BLBC cells (Figure 1). The results here showed that KLF5 promotes BLBC stemness partially through transcriptional regulation of *EphA2*.

KLF5 expression can be induced by many oncogenic and proinflammatory factors, such as TNF-α, lipopolysaccharide, and interleukin-1β^50^-^52^. In this study, TNF-α induced KLF5-EphA2 expression and increased the stemness of BLBC cells (Figure 3); interestingly, TNF-α promoted EphA2 expression and increased the level of EphA2 pS897, possibly by RSK1/2 activation^44^. Moreover, when the EphA2 inhibitor ALW-II-41-27 was used, the EphA2 pS897 level was dramatically inhibited, and the stemness of BLBC cells decreased (Figure 4). These results suggest that TNF-α promotes the stemness of BLBC through the KLF5-EphA2 axis to a certain extent. However, the downstream mechanism by which the TNF-α-KLF5-EphA2 axis and activated EphA2 pS897 increase BLBC stemness remains to be further studied.

In this study, we found that the knockdown of KLF5 or EphA2 could improve the drug sensitivity of BLBC cells to the chemotherapeutic agents PTX and DDP (Figure S4), suggesting that this may be related to KLF5- and EphA2-mediated BLBC stemness. Numerous studies have confirmed the close correlation between tumor cell stemness and chemoresistance in glioma, colorectal cancer, breast cancer, leukemia, and oral cancer^53^-^56^. In addition, studies have shown that knockdown of KLF5 could reduce cellular resistance to adriamycin in mesenchymal thyroid cancer, while high expression of KLF5 in colorectal cancer predicted poorer neoadjuvant chemotherapy outcomes^26^. Crosstalk between KLF5 and Hippo factors in breast cancer have been reported^57^-^60^. EphA2 drives chemotherapy resistance in gastric cancer cells by stabilizing YAP^61^. Thus, it is an interesting question whether hippo pathway is also involved in EphA2 regulation or function. The ERK1/2-RSK1/2-EphA2-GPRC5A signaling axis induced by cisplatin and carboplatin chemotherapy in serous ovarian carcinoma cell lines is closely correlated with acquired chemoresistance in cancer cells^62^. It will be investigated whether pS897 EphA2-induced chemosensitivity is mediated by drug-resistance gene expression in breast cancer stem cells.

Studies have shown that EphA2 pS897 activates the AKT, STAT3, SOX-2, and c-MYC signaling pathways and plays a crucial role in nasopharyngeal carcinoma stem cell formation^45^. Inhibition of EphA2 phosphorylation using the kinase inhibitor ALW-II-41-27 reduced the survival and proliferation of erlotinib-resistant lung cancer cells *in vitro* and *in vivo*^29^. In addition, EphA2 pS897 increased resistance to platinum-based chemotherapeutic agents in ovarian cancer^62^. However, there has been no report about whether there is a mechanism of phosphorylation of EphA2 at the S897 site promoting BLBC cell stemness and thereby enhancing chemoresistance. From this study, we found that low doses of PTX and DDP promoted the phosphorylation of EphA2 pS897 and increased the stemness of BLBC cells. We also found that ALW-II-41-27 suppressed the stemness of BLBC cells (Figure 4 and Figure 5). The use of low doses of the inhibitor ALW-II-41-27 reduced the IC50 of BLBC cells to the chemotherapeutic agents PTX and DDP. It was also confirmed that the administration of low doses of the inhibitor ALW-II-41-27 significantly enhanced the killing effect of low-dose PTX and DDP on tumor growth, remarkably, with no significant toxic side effects. In addition, high expression of EphA2 pS897 in BLBC patients may be associated with a poorer NCT effect (Figure 6). These results suggest that phosphorylation of EphA2 at S897 plays an important role in chemoresistance in BLBC. In conclusion, we confirmed that the KLF5-EphA2 axis may serve as a new target for the treatment of BLBC.

Although accumulated evidence supports the targeted treatment of EphA2 for cancer therapy, challenges remain. ANXA1 is reported as a positive regulator of EphA2 in Nasopharyngeal Carcinoma, gastric and colon cancer, targeting which can decrease EphA2 protein to suppress the cancer cells^63^, ^64^. Increasing evidence showed ANXA1 is regarded as a precision medicine for triple-negative breast cancer, however, no ANXA1-EphA2 axis was reported in breast cancer. Interestingly, we found a high correlation on mRNA expression of *ANXA1* and *EphA2* in breast cancer patients (data not shown), indicating a possible regulation of ANXA1 could contribute to high level of EphA2, beside our mechanism reported here. The expression and function of EphA2 in multiple cells and tissue types represent both an opportunity and a challenge. Inhibition of EphA2 in normal tissues while fighting cancer may lead to unintended toxic effects. In addition, the multiple signaling patterns of the EphA2 receptor complicate targeting strategies, and the most appropriate approach may vary from tumor to tumor. Overall, the important role of EphA2 in oncobiology makes it a promising therapeutic target, and ongoing clinical efforts for EphA2 may provide a valuable new weapon in the fight against BLBC.

## Materials and methods

### Cell culture and chemical drugs

All cell lines used in this study were purchased from the American Type Culture Collection (ATCC) and validated by short tandem repeat (STR) analysis. The BLBC cell lines HCC1806 and HCC1937 were cultured in RPMI-1640 medium (Gibco, C11875500BT) with 5% fetal bovine serum (FBS) (ExCell Bio, FSD500). HEK293T cells were cultured in DMEM (Gibco, C11995500BT) with 10% FBS. All cells were maintained in an incubator at 37°C with 5% CO_2_. Drugs and cell factors included ALW-II-41-27 (TopScience, T4344), DDP (Innochem, HY-17394), PTX (Sigma‒Aldrich, T7402), and TNF-α (SinoBiological, 10602-HNAE).

### Cell transfection and production of lentiviral particles

siRNA or plasmid DNA was transfected using Lipofectamine® 2000 Reagent (Invitrogen, 11668-019) according to the manufacturer's instructions. Lentivirus packaging and infection: For knockdown experiments, lentiviruses were generated using pSIH and packaging plasmids (psPAX2 and pMD2g) in HEK293T cells; for overexpression experiments, lentiviruses were generated using pLVX-Puro and packaging plasmids (psPAX2 and pMD2g) or pCDH-3×Flag and packaging plasmids (psPAX2 and pMD2g) in HEK293T cells. Lentiviruses were collected at 48 h and then stored at -80°C. Lentiviruses were added to HCC1806 and HCC1937 cells with 8 μg/mL polybrene (Sigma, H9268). After 24 h, the medium was replaced with fresh medium supplemented with 1 μg/mL puromycin (InvivoGen) to select stably infected cells.

### Quantitative reverse transcription-polymerase chain reaction (RT‒qPCR)

Total RNA was isolated using TRIzol reagent (Invitrogen, 15596026), and then 1 μg of total RNA was reverse transcribed to cDNA according to the manufacturer's instructions for the HiScript II Q RT SuperMix for qPCR (+gDNA wiper) kit (Vazyme, R223-01). Quantitative PCR was performed on the 7900HT Fast Real-Time PCR System (Applied Biosystems, 4351405) using SYBR Green reagents (ABI, Austin, TX, CA, USA). The primer sequences used are listed in Supplementary Table 1.

### Western blotting

For immunoblotting, cells were harvested, washed with ice-cold 1×PBS buffer, lysed in RIPA buffer supplemented with 1×Proteinase Inhibitor Cocktail (MCE, HY-K0010), and centrifuged at 12,000 rpm at 4°C for 10 min. Protein lysates were fractionated by 10% SDS polyacrylamide gel electrophoresis and then transferred to polyvinylidene fluoride (PVDF) membranes (Merck Millipore, #IPFL00010, Germany). The membranes were blocked with 5% nonfat milk for 1 h at room temperature and then incubated with the indicated primary antibodies (KLF5, R&D system AF3758; EphA2, Santa Cruz sc-398832; pS897 EphA2, Affinity AF7279; GAPDH, Santa Cruz sc-32233; β-actin, Sigma A5441) overnight at 4°C, washed, and probed with HRP-conjugated secondary antibodies (Invitrogen, #31460 & #31430). Finally, an HRP substrate kit (US Everbright, S6009 L) and ECL Detection System of ImageQuant LAS 4000 were used for signal detection.

### Dual luciferase assays

The DNA fragments of the EphA2-WT promoter and EphA2-Mutant promoter were cloned into the pGL3-Basic vector. A dual-luciferase reporter assay kit (Promega) was used to measure the luciferase activity according to the manufacturer's instructions. Luciferase activity was normalized by using a Renilla luciferase internal control. The primers used are listed in Supplementary Table 1.

### Chromatin immunoprecipitation and ChIP‒qPCR

ChIP assays were performed using the BLBC cell lines HCC1937 and HCC1806 following the manufacturer's instructions (Abcam, Cambridge, MA, USA). Protein A/G beads (MCE, HY-K0202-1) were first mixed with an equal amount of anti-KLF5 antibody (R&D system, AF3758) or rabbit IgG (Proteintech) and incubated overnight at 4°C. HCC1937 and HCC1806 cells were fixed with 1% formaldehyde (Sigma) for 15 min at room temperature (RT). Glycine (125 mM) was added to quench the formaldehyde and terminate the cross-linking reaction. The cells were scraped into an Eppendorf (EP) tube and centrifuged at 500×g at 4°C for 10 min. The supernatant was aspirated off, and the pellet was resuspended in cytoplasmic lysis buffer (5 mM PIPES pH 8.0, 85 mM KCl, 0.5% Nonidet P-40 with protease inhibitors; 1000 μL per 1×10^7^ cells). The cell suspension was centrifuged at 4000×g for 5 min at 4°C. Finally, the pellet was resuspended in nuclear lysis buffer (50 mM Tris-HCl pH 8.1, 10 mM EDTA, 1% SDS with protease inhibitors; 500 μL per 1×10^7^ cells) and then sonicated for ten cycles, with 30 s on and 30 s off for each cycle. The DNA‒protein complex was mixed with the antibody-A/G bead complex and incubated at 4°C overnight. The chromosomal DNA was purified by phenol/chloroform extraction and ethanol precipitation. The pellets were dissolved in 100 μL of ddH_2_O for qPCR. Primers are listed in Supplementary Table 1.

### Cell proliferation

Cell proliferation was assessed by sulforhodamine B (SRB) assays to measure cell proliferation and viability.

### Flow cytometry

The ALDEFLUOR Assay Kit (#01700; Stemcell Technologies, Vancouver, BC, Canada) was used for the detection of ALDH^+^ cells by flow cytometry. Approximately 1×10^6^ cells were resuspended in 1 mL assay buffer. Then, the sample was added to an activated Aldecount Reagent tube. Subsequently, 0.5 mL of cells were immediately placed into a new tube with 5 μL of DEAB buffer and then gently mixed. All samples were incubated for 45 min at 37°C in the dark and centrifuged at 500×g for 5 min. The cells were then resuspended in 0.5 mL assay buffer and analyzed immediately by flow cytometry. All representative images for flow cytometry analysis for ALDH^+^ or CD44^+^/CD24^-^ were showed as Supplementary Figure S5-S12.

### Mammosphere culture

A Mammosphere Culture Kit (#05620; Stemcell Technologies) was used for the mammosphere assay. To induce sphere formation, BLBC HCC1937 cells were digested into single cells and plated into 24-well ultralow attachment plates (#3473, Corning) at a density of 2000-5000 cells per well. The cells were cultured with 500 μL of complete MammoCult™ medium (#05620; Stemcell Technologies). After 14 days, the number and size of mammospheres were assessed.

### A limiting dilution assay for tumorigenesis

The BLBC cell line HCC1806 (2×10^4^, 2×10^5^, 2×10^6^) was suspended in RPMI-1640 serum-free medium and Matrigel (#354234, Corning, BD Biocoat) at a 1:1 ratio and injected into the fat pads of 6- to 7-week-old female BALB/c nude mice from the Hunan SJA Laboratory Animal Co., Ltd. (Changsha, Hunan, China). After 4-5 days, the site of implantation was monitored for tumor growth, and tumor size was measured every 2 or 3 days. Mice were randomly assigned to control and experimental groups without investigator blinding. Finally, all the mice were sacrificed, and the tumors were collected for analysis. This animal experimentation was approved by the animal ethics committee of Kunming Institute of Zoology, CAS.

### Animal experiments

Six- to seven-week-old female BALB/c nude mice were purchased from SJA Laboratory Animal Co., Ltd. (Changsha, Hunan, China). The animal protocol was approved by the animal ethics committee of Kunming Institute of Zoology, CAS. Nude mice were randomly distributed into six groups (n=5 for each group, with two sites per mouse). HCC1806 cells (1×10^6^) were resuspended in Matrigel (#354234, Corning, BD Biocoat; 1:1 diluted with RPMI-1640 serum-free medium) and injected into the fourth pair of mammary gland fat pads. Tumor sizes were measured every 4 days, and the tumor volume was calculated using the following equation: volume = (length×width^2^)/2. When the maximum diameter of the tumor reached approximately 14 mm, all the mice were sacrificed, and the tumors were collected for analysis.

### Clinical samples

Breast cancer samples were collected from the Third Affiliated Hospital of Kunming Medical University in Yunnan. Treatment information for neoadjuvant chemotherapy samples is listed in Supplementary Table 2. The research was approved by the Institute Research Ethics Committee of the Third Affiliated Hospital of Kunming Medical University and was conducted in strict accordance with the International Ethical Guidelines for Biomedical Research Involving Human Subjects (CIOMS) ethical guidelines (KYCS2022082). The samples were mainly used for immunohistochemistry staining.

### Immunohistochemistry (IHC)

Briefly, the samples were fixed with 4% formaldehyde for 48 h at room temperature and embedded in paraffin. Then, the paraffin-embedded tissue sections at 5-8 μm thickness were transferred onto glass slides. The slides were deparaffinized, rehydrated, and pressure cooker heated for 2-5 min in EDTA for antigen retrieval. Endogenous peroxidase activity was inactivated by adding an endogenous peroxidase blocker (OriGene, Beijing, China) for 15 min at room temperature. Slides were incubated overnight at 4°C with KLF5 (Proteintech, 66850-1-Ig), EphA2 (Santa Cruz, sc-398832), and EphA2 pS897 (Cell Signaling, #6347) antibodies. Next, the slides were washed three times with 1×PBS and incubated with secondary antibody (OriGene, Beijing, China) at room temperature for 20 min, DAB concentrate chromogenic solution (1:200 dilution of concentrated DAB chromogenic solution), counterstained with 0.5% hematoxylin, dehydrated with graded concentrations of ethanol for 3 min each (70%-95%-100%), and finally stained with dimethyl benzene. Immunostained slides were evaluated by light microscopy. IOD score: integrated optical density score.

### Statistical analysis

GraphPad Prism software was used to perform statistical analysis in this study. All experiments were repeated three times. All data are shown as the mean ± SD, and differences were analyzed using Student's t test. P values less than 0.05 were considered significant. *** or ###: *P* < 0.001, ** or ##: *P* < 0.01, * or #: *P* < 0.05, ns, not significant, t test.

## Supplementary Material

Supplementary figures and tables.
