# Supplementary material for: Targeting the KLF5-EphA2 axis can restrain cancer stemness and overcome chemoresistance in basal-like breast cancer
Source: Int J Biol Sci. 2023 Mar 21;19(6):1861–74. doi: 10.7150/ijbs.82567 (PMC10092769; doi:10.7150/ijbs.82567)
Supplement: Supplementary file 1 — Supplementary figures and tables. [file ijbsv19p1861s1.pdf]

Supplementary Data for:

Targeting the KLF5-EphA2 axis can restrain cancer stemness and overcome chemoresistance in basal like breast cancer

Ping Zhao[1,#], Jian Sun[1,#], Yiyin Tang[1,#], Xiangwu Zhang[1], Xin Liu[1], Rong Liu[3], Guangshi Du[4], Wenqiang Gan[2], Chuanyu Yang[2], Ceshi Chen[1,2,5]*, Dewei Jiang[2,6]*

1 *The Third Affiliated Hospital, Kunming Medical University, Kunming, 650118 China*
2 *Key Laboratory of Animal Models and Human Disease Mechanisms of the Chinese Academy of Sciences and Yunnan Province, Kunming Institute of Zoology, Chinese Academy of Sciences, Kunming, 650201 China*
3 *Translational Cancer Research Center, Peking University First Hospital, Beijing, 100034 China*
4 *Translational Medicine Research Center, Guizhou Medical University, Guiyang, 550025 China*
5 *Academy of Biomedical Engineering, Kunming Medical University, Kunming, 650500 China*
6 *Kunming College of Life Sciences, University of the Chinese Academy of Sciences, Kunming, 650204 China*

This file includes:

- ✧ **Supplementary Figures S1-S12**
- ✧ **Supplementary Tables 1-2**

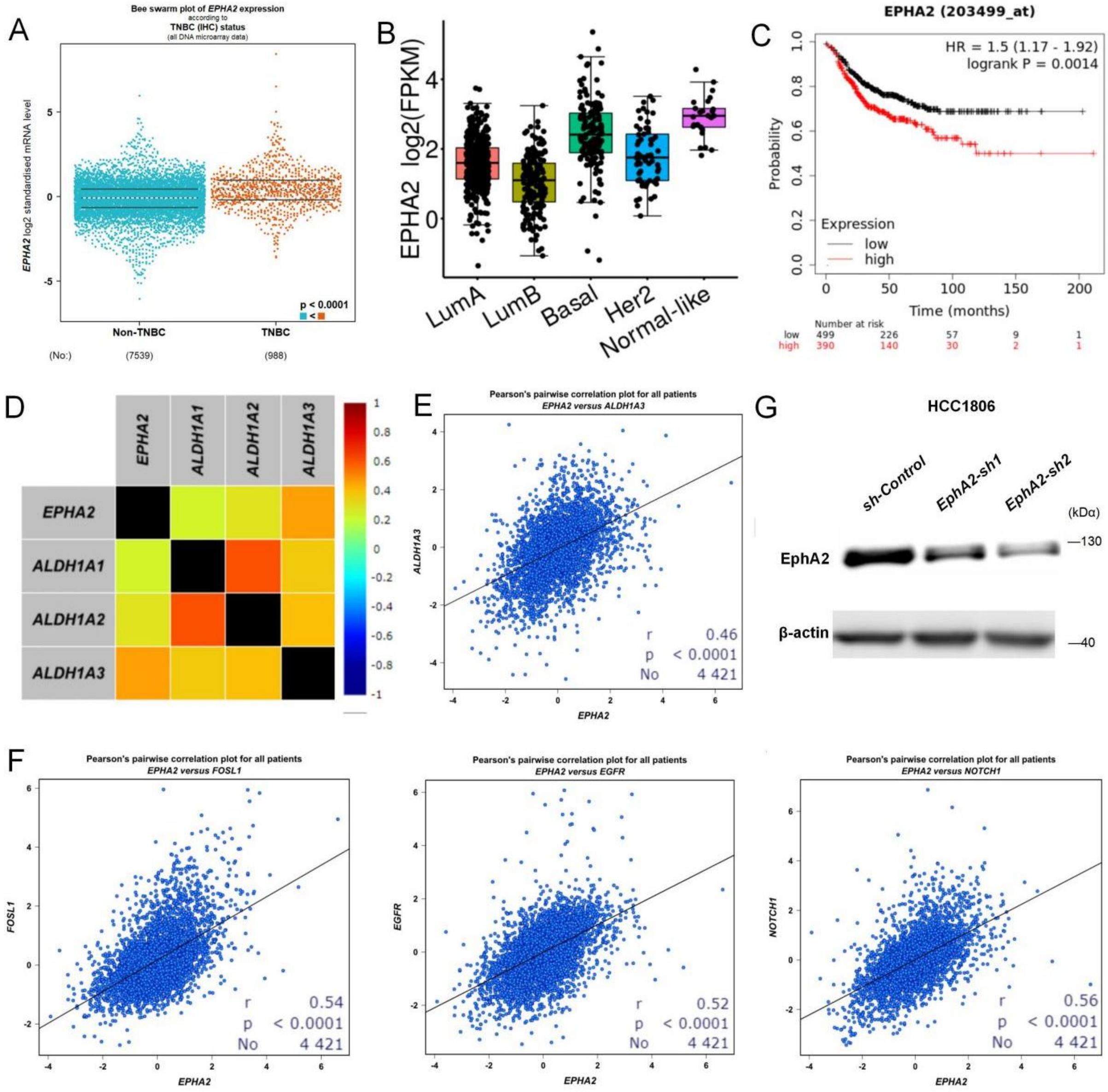

Figure S1. EphA2 is associated with poor prognosis and breast cancer cell stemness in breast cancer patients.

A. According to database analysis (bc-GenExMiner v4.9), EphA2 was found to be highly expressed in basal-like type patients and expressed at low levels in non-basal-like type patients; B. TCGA database analysis revealed that the mRNA level of *EphA2* was highly expressed in basal-like type patients; C. High expression of EphA2 was positively correlated with poor prognosis of breast cancer patients (Kaplan Meier-Plotter); D-E. The expression level of EphA2 was positively correlated

with ALDH1A1-3 (bc-GenExMiner v4.9); F. The expression level of *EphA2* was positively correlated with *FOSL1*, *EGFR*, and *NOTCH1* (bc-GenExMiner v4.9); G. WB validation of stable knockdown of EphA2 in HCC1806 cells by two independent shRNAs.

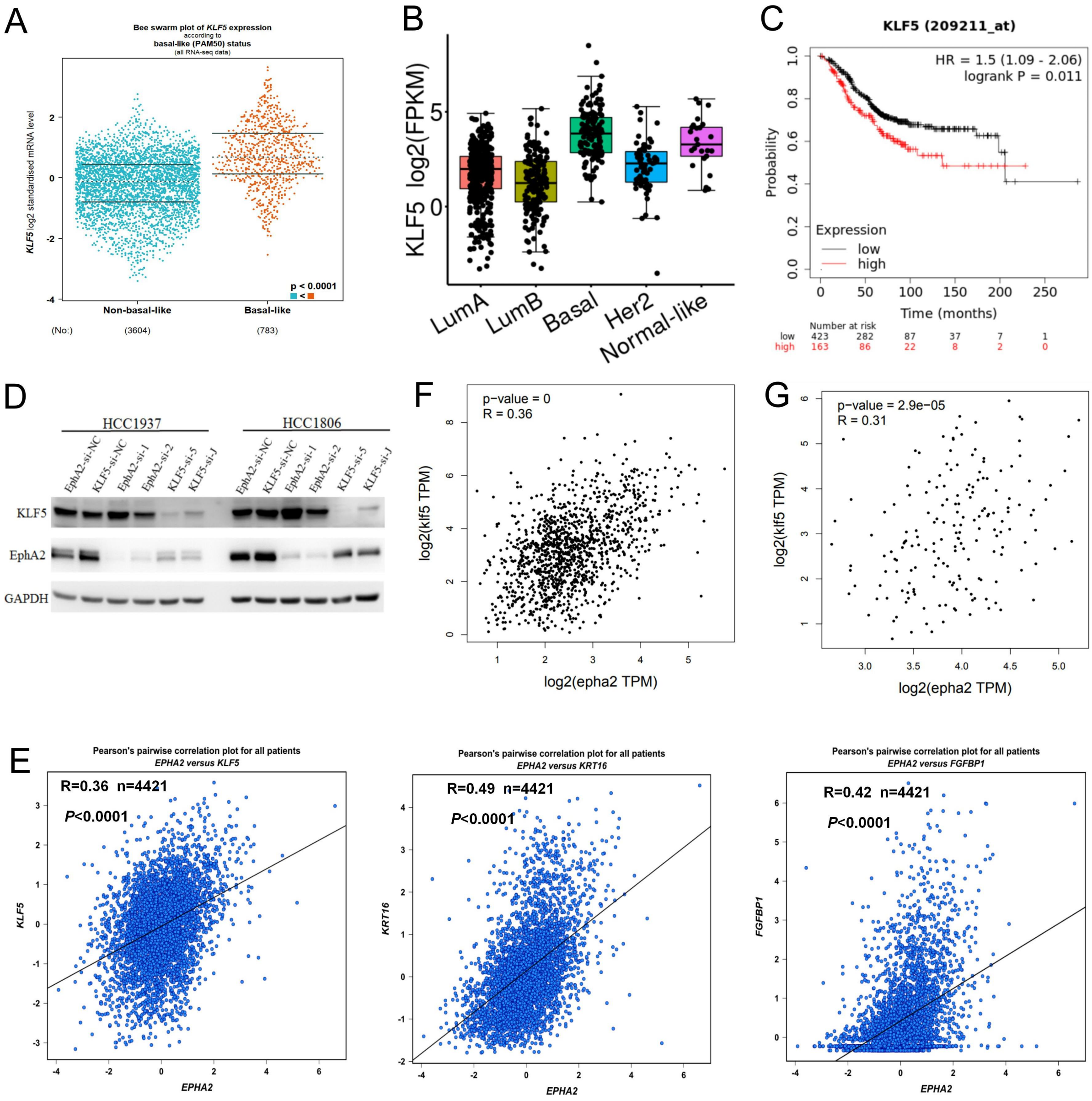

Figure S2. Positive correlation between EphA2 and KLF5 expression in breast cancer.

A. According to database analysis (bc-GenExMiner v4.9), KlF5 was found to be highly expressed in basal-like type patients and expressed at low levels in non-basal-like type patients; B. TCGA database analysis revealed that the mRNA level of *KLF5* was highly expressed in basal-like type patients; C. High expression of KLF5 was positively correlated with poor prognosis of high-grade breast cancer patients (Kaplan Meier-Plotter); D. The knockdown of KLF5 in HCC1937 and

HCC1806 could inhibit the expression of EphA2, but the knockdown of EphA2 did not affect the expression of KLF5; E. In the analysis of all breast cancer patients in the database (bc-GenExMiner v4.9, n=4421), it was found that the expression trends of EphA2 and KLF5, KRT16, and FGF-BP1 were consistent, all positively correlated (R=0.36, 0.49, and 0.42, respectively); F-G. EphA2 was positively correlated with KLF5 expression in the TCGA (F) and GTEx (G) databases.

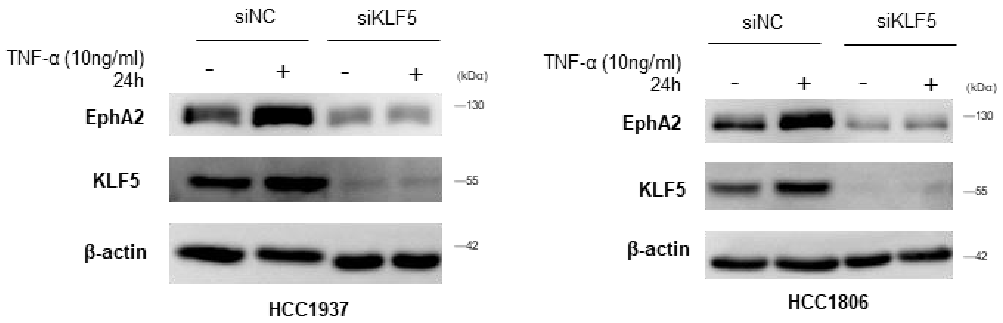

Figure S3. Knockdown of KLF5 inhibits TNFα-induced EphA2 expression

Knockdown of KLF5 in HCC1937 (left) and HCC1806 (right) cells with the addition of TNFα for detection of EphA2 and KLF5 expression by WB.

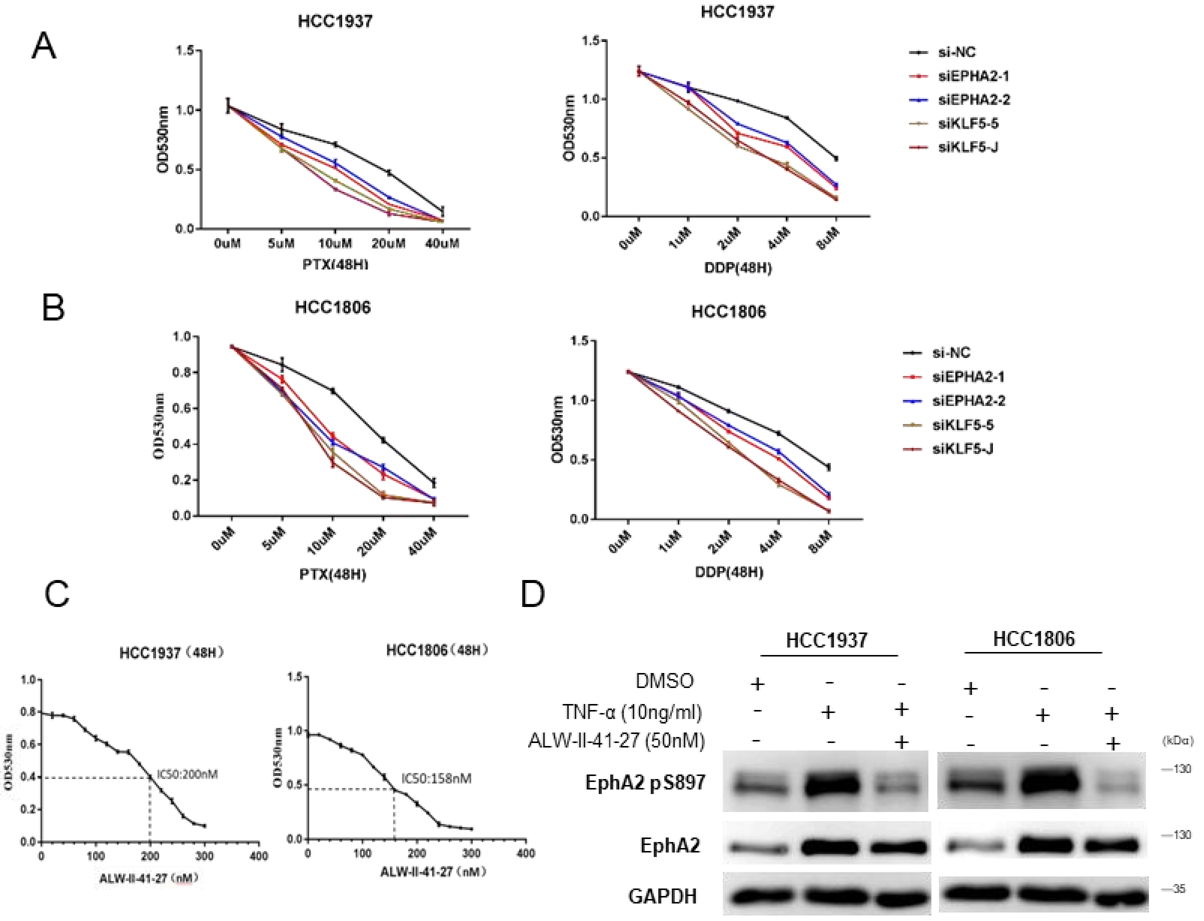

Figure S4. ALW-II-41-27 inhibited BLBC cells.

A-B. Detection of tolerance to PTX and DDP after knockdown of *EphA2* or *KLF5* in HCC1937 (A) and HCC1806 cells (B), respectively; C. Determination of IC50 of HCC1937 and HCC1806 cells against ALW-II-41-27 by SRB assays; D. Detection of expression of EphA2 and EphA2 pS897 by WB after treatment of HCC1937 and HCC1806 cells with TNFα (10 ng/mL) alone or in combination with ALW-II-41-27 (50 nM) for 24 h.

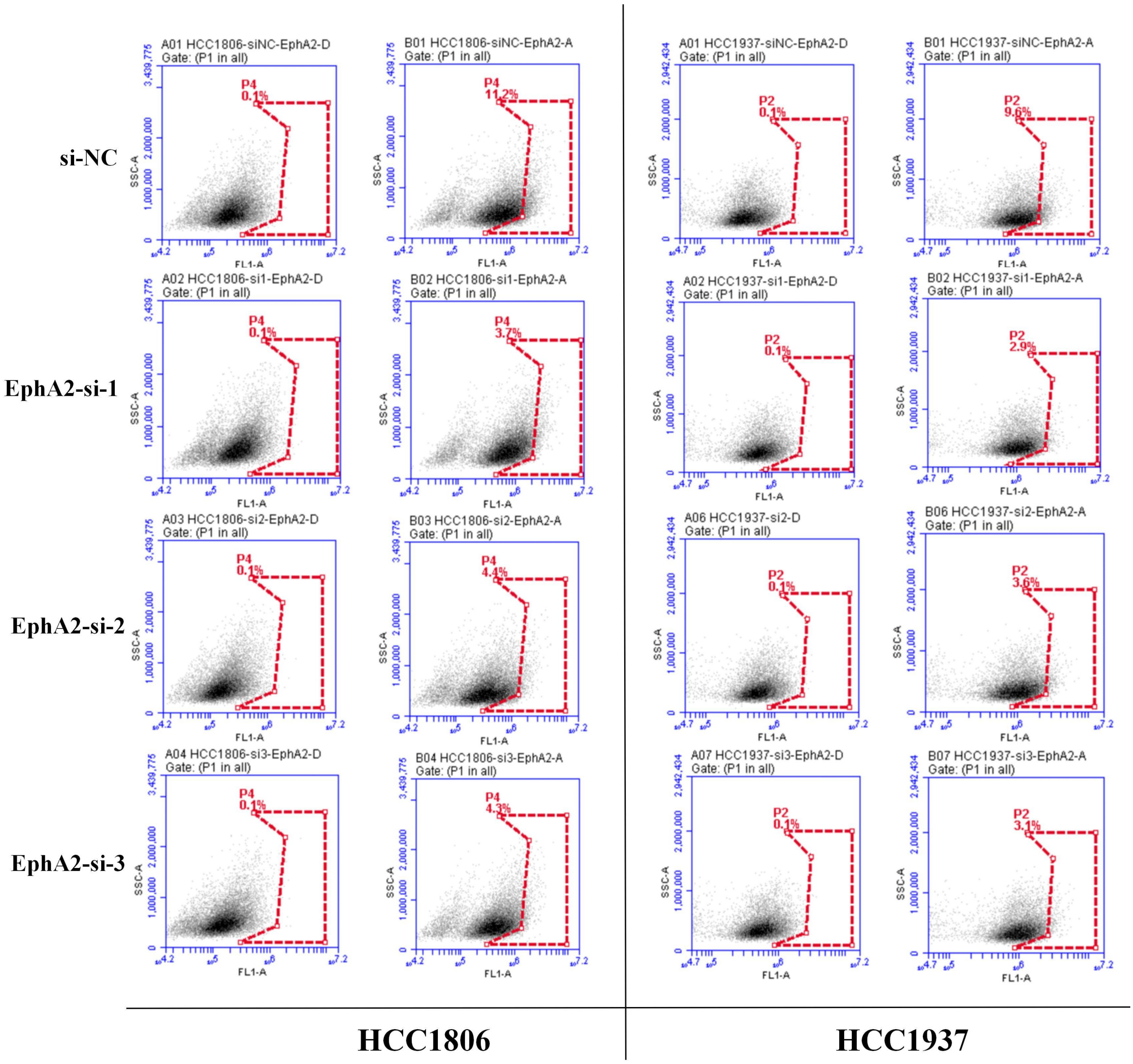

Figure S5. Representative images for flow cytometry analysis for Figure 1D-E.

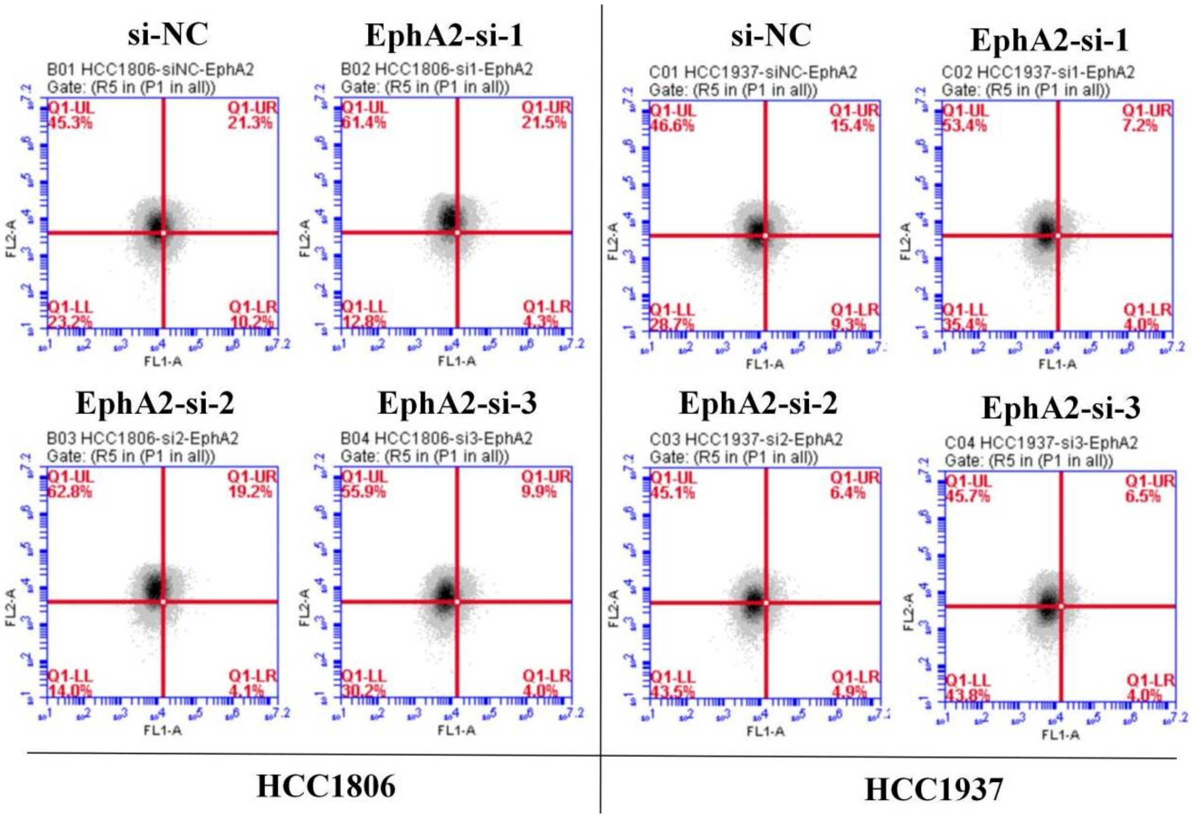

Figure S6. Representative images for flow cytometry analysis for Figure 1F-G.

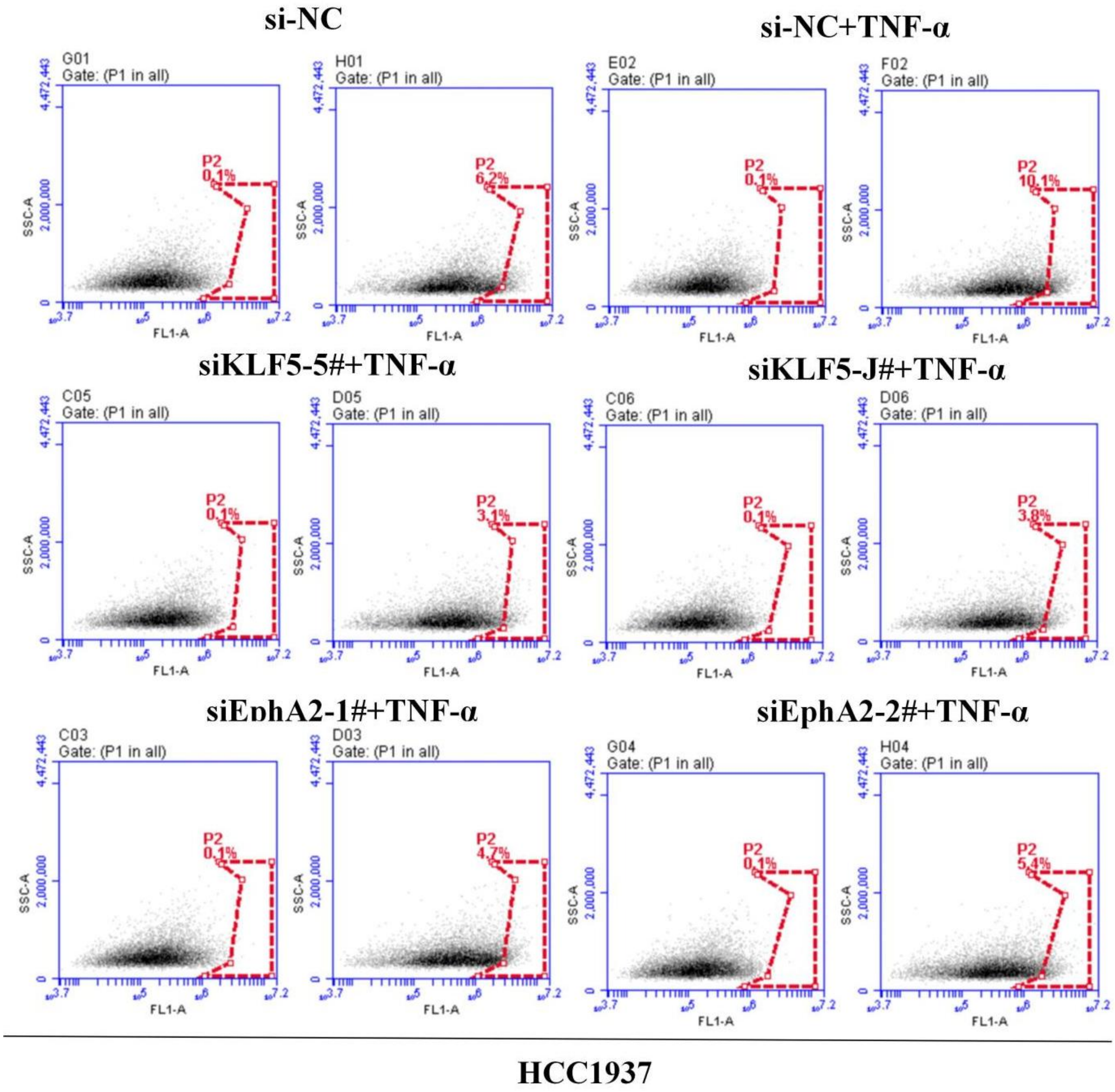

Figure S7. Representative images for flow cytometry analysis for Figure 3B.

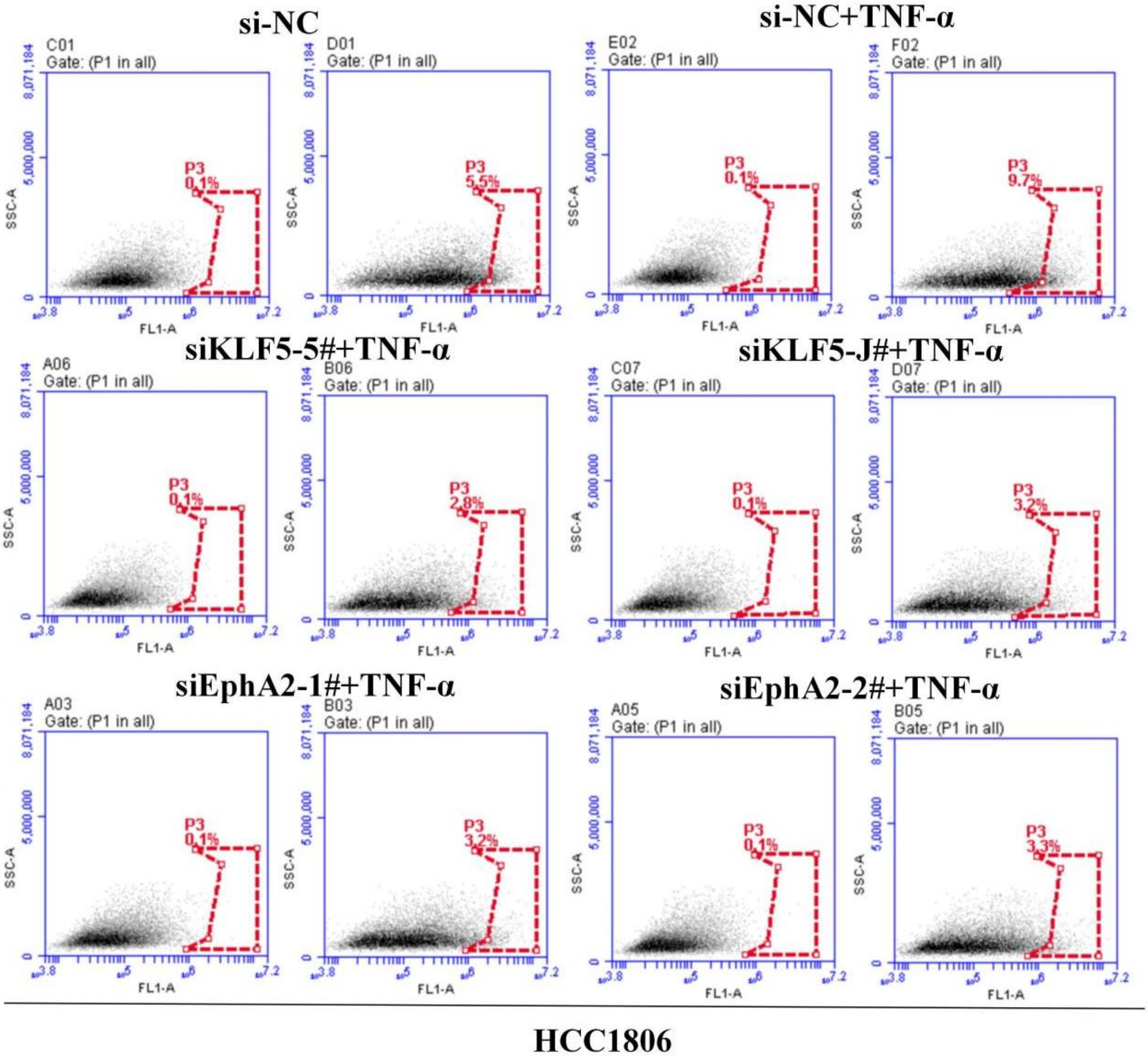

Figure S8. Representative images for flow cytometry analysis for Figure 3C.

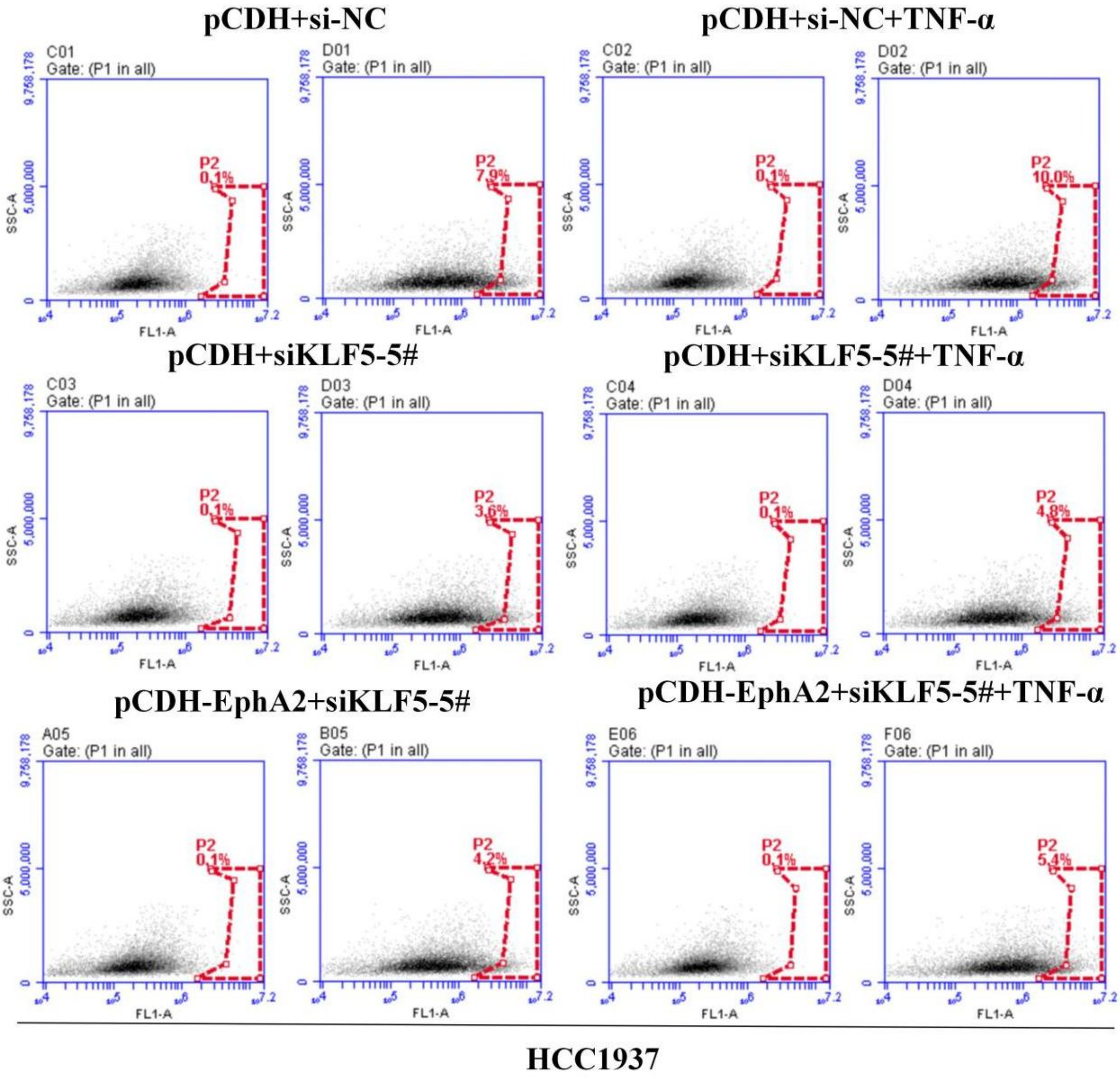

Figure S9. Representative images for flow cytometry analysis for Figure 3G.

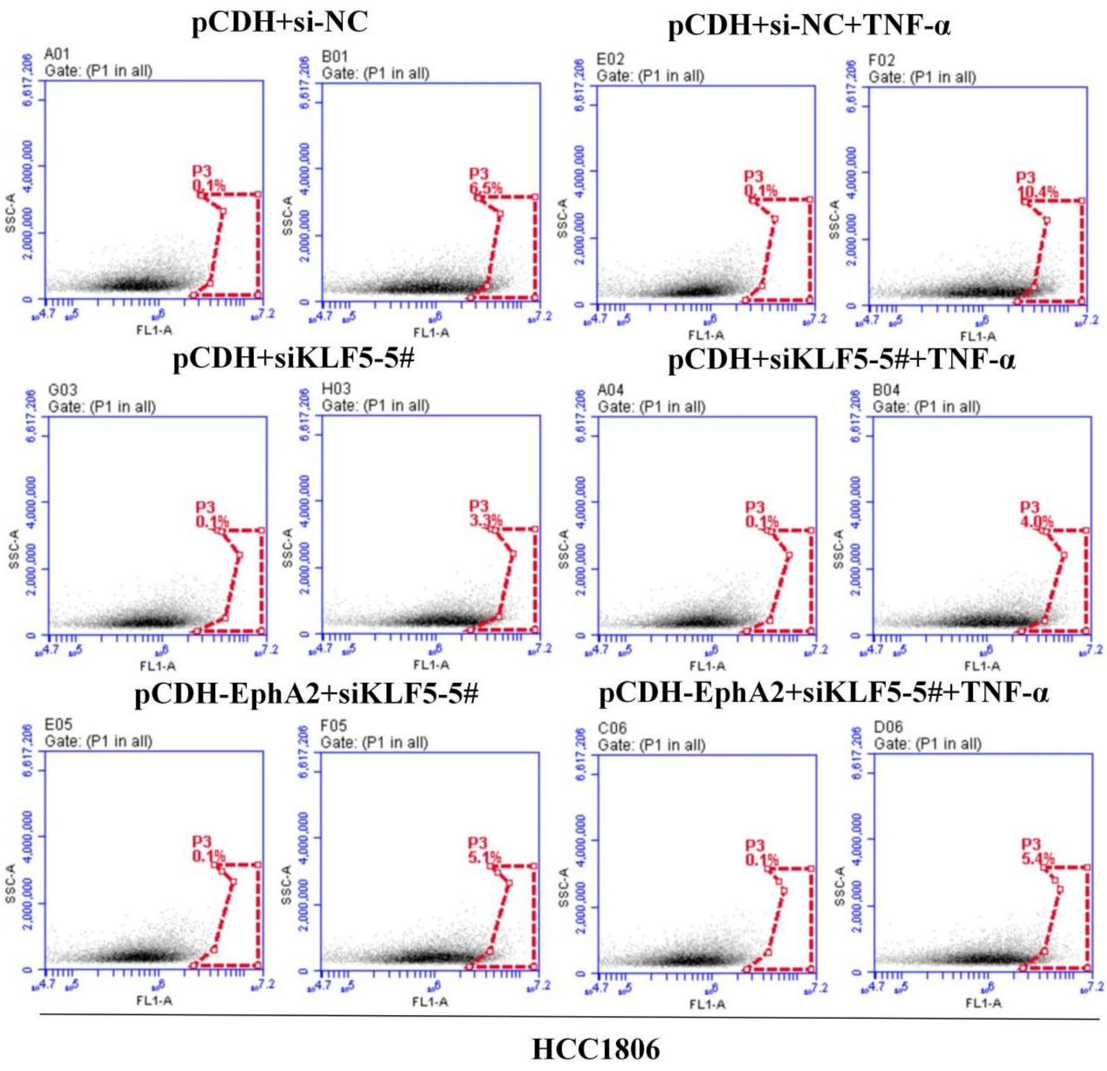

Figure S10. Representative images for flow cytometry analysis for Figure 3H.

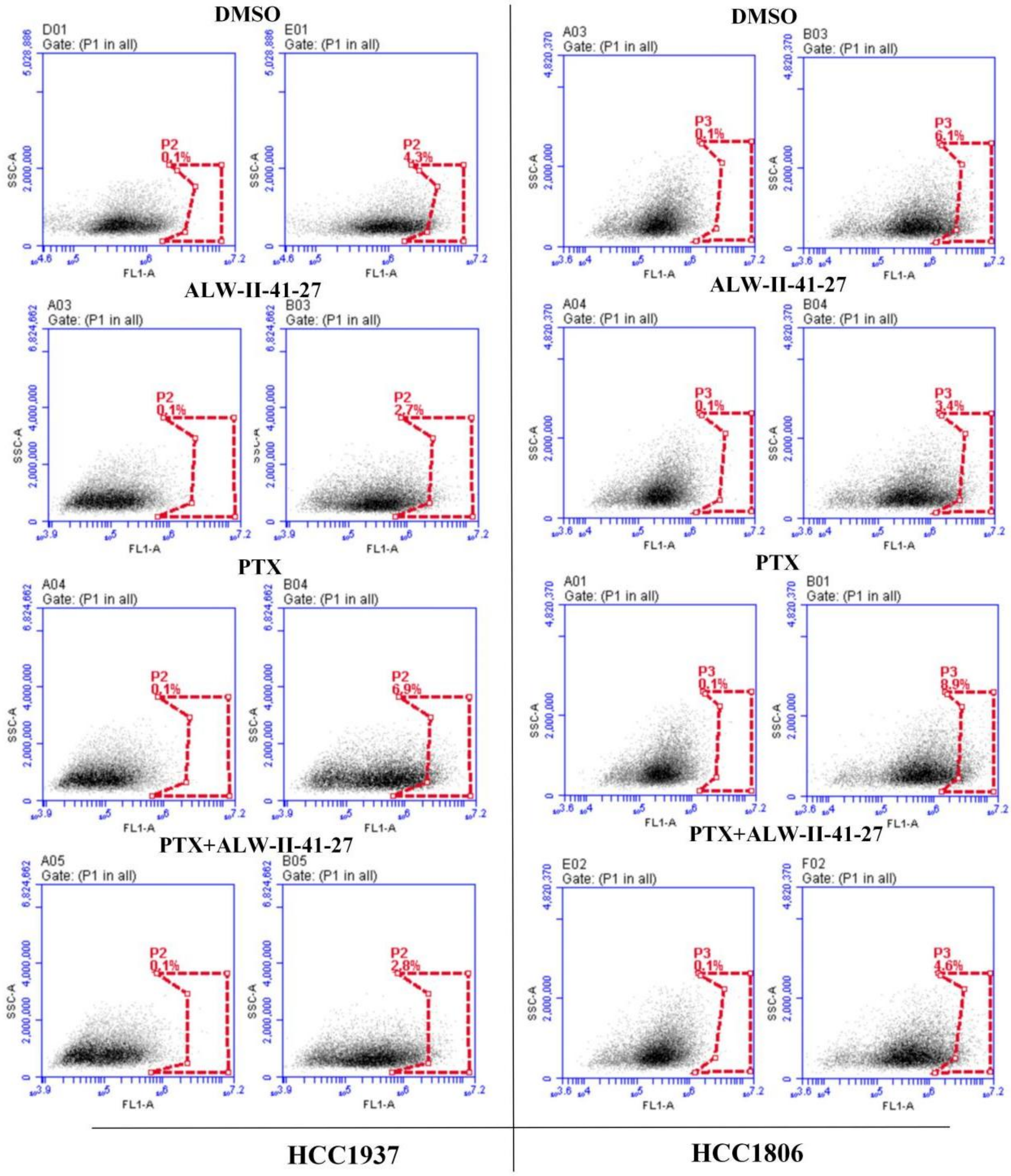

Figure S11. Representative images for flow cytometry analysis for Figure 4C.

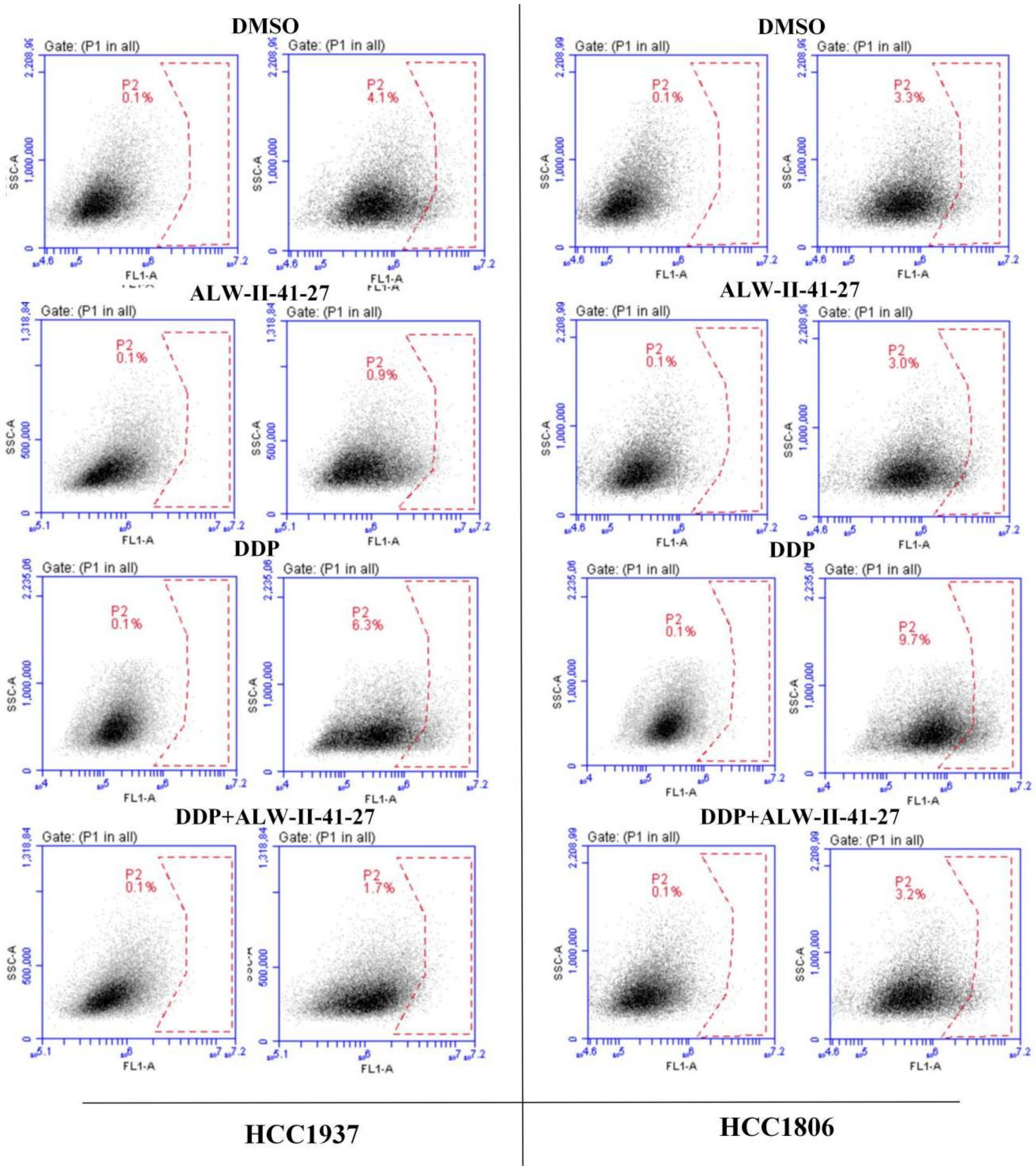

Figure S12. Representative images for flow cytometry analysis for Figure 4D.

Supplementary Tables

Table S1. Sequences of primers used in this paper.

Primers name	Sequences (5'-3')
EphA2 promoter WT forward primer	CCGTACCTCCGTCGGAGACCTCACCCTCCGCCCTC
EphA2 promoter WT reverse primer	ACTGAGACACAGGGCGATTG
EphA2 promoter MT forward primer	CCGTACCTCCGTCGGAGACCTCACTTCTTATTTCC
EphA2 promoter MT reverse primer	ACTGAGACACAGGGCGATTG
P1 forward primer	GGGCCCCACAATAGGTATCC
P1 reverse primer	AACATTGGCTTCCGTTCCCT
P2 forward primer	TTTTCCAAGGAACCTGCCTC
P2 reverse primer	CCAGAATTGAGGGGCATGGG
Negative forward primer	TTAAGGACTCGGGGCAGGAG
Negative reverse primer	ATCAGGTCCCACTTCCTTGC
EphA2 forward primer	GCAAAGGGTGGGACCTGATG
EphA2 reverse primer	TTGGTGCGGAGCCAGTTGT
GAPDH forward primer	CGACACCCACTCCTCCACCTT
GAPDH reverse primer	CCACCACCCTGTTGCTGTAGCC
EphA2-siRNA1	GCAGCAAGGTGCACGAATT
EphA2-siRNA2	GCTCAAGTTTACTGTACGT
EphA2-siRNA3	GCAGGAGTTGGCTTCTTTA
EphA2-shRNA1-F	CCGGGCCAGTTTAGCCACCACAATACTCGAGTATTGTGGTGGCTAAACTGGCTTTTT
EphA2-shRNA1-R	AATTCAAAAAGCCAGTTTAGCCACCACAATACTCGAGTATTGTGGTGGCTAAACTGGC
EphA2-shRNA2-F	CCGGCGAGGTCATGAAAGCCATCAACTCGAGTTGATGGCTTTCATGACCTCGTTTTT
EphA2-shRNA2-R	AATTCAAAAACGAGGTCATGAAAGCCATCAACTCGAGTTGATGGCTTTCATGACCTCG

Table S2. Clinical information for patients' response to NCT

BLBC（NCT）	chemo-sensitive<br><br>(N=19)	chemo-resistant<br><br>(N=11)
Miller Payne 1 or PD	0	11
Miller Payne 4-5 or PCR	19	0
Metastasis or recurrence（3 months）	0	4
Used docetaxel	19	11
Used cisplatin	3	7
Used anthracycline	19	11
Used cyclophosphamide	16	9
